# Clinicians and individuals with acquired brain injury perspectives about factors that influence mobility: creating a core set of mobility domains among individuals with acquired brain injury

**DOI:** 10.1080/07853890.2021.2015539

**Published:** 2021-12-13

**Authors:** Rehab Alhasani, Dennis Radman, Claudine Auger, Anouk Lamontagne, Sara Ahmed

**Affiliations:** a Faculty of Medicine, School of Physical and Occupation Therapy, McGill University, Montreal, Canada; b Centre for Interdisciplinary Research in Rehabilitation of Greater Montreal (CRIR), Montreal, Canada; c Department of Rehabilitation Sciences, College of Health and Rehabilitation Sciences, Princess Nourah Bint Abdulrahman University, Riyadh, Saudi Arabia; d Faculty of Medicine, School of Rehabilitation, Université de Montréal, Montreal, Canada; e Institut Universitaire sur la Réadaptation en Déficience Physique de Montréal, CIUSSS du Centre-Sud-de-l'Île-de-Montréal, Montreal, Canada; f Jewish Rehabilitation Hospital, CISSS de Laval, Laval, Canada; g Constance Lethbridge Rehabilitation Center, CIUSSS Centre-Ouest de l'Îile de Montreal, Montreal, Canada; h McGill University Health Center Research Institute, Clinical Epidemiology, Center for Outcome Research and Evaluation (CORE), Montreal, Canada

**Keywords:** Mobility, acquired brain injury, international classification of functioning, health and disability framework, focus group, assessment

## Abstract

**Objectives:**

To identify factors which may influence mobility and could be considered during the evaluation of mobility in individuals with acquired brain injury (ABI) following qualitative focus groups with both clinicians and individuals with ABI, to assess their needs and preferences in order to individualize their care management plans.

**Methods:**

Five focus groups were held, three with clinicians from 3 rehabilitation sites of CRIR (CRDM: *n* = 4; IURDPM: *n* = 3; JRH: *n* = 10) and two with individuals with ABI from one rehabilitation site (CRDM) (individuals with stroke: *n* = 5; individuals with TBI: *n* = 5). Focus group discussions were transcribed and analyzed using inductive and deductive thematic content approaches.

**Results:**

Four themes were identified: considering mobility holistically and individual needs, preferences, and unique experiences; assessment and intervention guidelines; support network; and uncertainty about symptoms and recovery. Using the ten-rule International Classification, Functioning, Disability, and Health framework linking process, codes were categorized into Body Functions Activity and Participation, and Environmental Factors exploring the prominent domains that mostly identify factors influencing mobility.

**Conclusions:**

Comprehensive measurement of mobility remains an ongoing challenge owing to multiple contributing factors, ranging from personal and psychosocial factors to the influence of a myriad of environmental and community considerations. Preparing individuals with ABI for community mobility can be substantially improved if healthcare professionals employ communicative tools to facilitate shared decision making with patients and to deliver patient-centred rehabilitation care.

## Introduction

Acquired Brain Injury (ABI), including traumatic brain injury (TBI) and stroke, are the leading causes of disability globally [1–3]. According to the World Health Organization, the global incidence of all-severity TBI is estimated at 69 million people, while 15 million people suffer a stroke worldwide each year [4–6]. Among the 1.5 million Canadians with ABI, over 60% report ongoing restrictions in mobility and participation in societal roles [5]. Individuals with ABI face challenges, especially once discharged from acute care or rehabilitation and with uncertainty regarding recovery and regaining independence [7,8]. Mobility limitations in the community are common [9] and affects 30% of persons with a TBI [10–12], and up to 50% of stroke survivors [13], even after extensive rehabilitation [9]. Long-term follow-up of individuals with ABI show that impairments in mobility appear to undergo little change, even ten years after the initial injury [11,12,14]. Most individuals with ABI have decreased levels of community mobility, significantly reducing their quality of life [15]. Identifying effective strategies and interventions to mitigate the long-term consequences, management, and rehabilitation of people with ABI is a priority [16].

Mobility is a multidimensional construct defined through both theoretical and empirical approaches. Theoretically, mobility consists of the ability to move oneself independently within a ‘life-space’, expanding from one’s home to the neighbourhood and beyond [17–23]. Webber’s framework adds that mobility is influenced by five vital interrelated determinants, including physical, environment, cognition, psychosocial, and financial [23], and this broadness and complexity is reflected in the International Classification, Functioning, Disability, and Health framework (ICF) mobility core set [24]. Empirically, studies have focussed on the effects of the built environment on mobility within the community [25,26]. Also, studies based on the preceding frameworks showed that diagnosis alone is not enough to predict mobility limitations, and that length of hospitalization and intensity of care are needed to accurately predict return to work potential, work performance, or social integration [23–26]. Also, social and healthcare decision makers recognize that to decrease the incidence and severity of disability and enhance mobility and participation requires modifying features of the social and physical environment [24].

Selection of a suitable measure to evaluate mobility is critical to accurately characterize mobility limitations, to plan intervention objectives, and to monitor changes in mobility during rehabilitation for individuals with ABI [27]. Choosing a measure of mobility, however, can be challenging for clinicians, as mobility is multidimensional, owing to the complex interaction of bio-psychosocial factors. There is no comprehensive measure to evaluate the myriad of factors that influence mobility for individuals with ABI [28,29]. Further, to measure mobility in research, we rely on expensive laboratory technologies [30–32] and performance-based tools [33] that are burdensome in terms of setup, staff time for administration, and analysis. Notably, these tools may not be readily applied in “real-life” community contexts. Further, electronic platforms that can collect real-time patient-reported and clinician-reported data are in their early stages [34], particularly in rehabilitation. To build these platforms correctly, a common language of the information collected in these systems is important to ensure that the data can be used to evaluate changes within and between patients. Therefore, to plan rehabilitation effectively and compare between different interventions, an understanding of the nature and severity of mobility among individuals with ABI is needed, which requires a comprehensive evaluation of mobility.

Comprehensive and accurate evaluation of mobility can help clarify differential benefits and harms of interventions. Measures that capture challenges in measuring mobility from clinicians and individuals with ABI perspectives are necessary during recovery, rehabilitation, and community reintegration. Identified factors that influence mobility can further inform clinicians on how to incorporate individual with ABI needs and preferences into individualized care management plans to generate health outcomes that matter most to patients.

## Objectives

To identify factors which may influence mobility and could be considered during the evaluation of mobility in individuals with acquired brain injury (ABI) following qualitative focus groups with both clinicians and individuals with ABI, to assess their needs and preferences in order to individualize their care management plans.

## Methods

### Statement of ethics

Approval of this study was granted by the Comité d’Éthique de la Recherche des Établissements du Centre de Recherche Interdisciplinaire en Réadaptation (CRIR) [CRIR 1387-1218].

### Research design, type of sampling, and data collection

Focus group discussions were selected as the best method to meet the aims of this study [35]. Focus groups are useful methodology to obtain information on perspectives and experiences of a homogenous group of people related to a common topic [36], as they facilitate discussion and produce a variety of ideas in a short time among participants [37,38]. Data collection took place at three rehabilitation sites of Centre for Interdisciplinary Research in Rehabilitation of Greater Montreal (CRIR) in the province of Quebec, Canada.

Pre-recruitment of individuals with ABI was accomplished using a computer-generated random list of previous rehabilitation clients in the sites since November 2019 using the following eligibility criteria: age ≥18 years, men or women with a primary diagnosis of stroke or TBI, files currently open or discharged ≤6 months, ability to speak French or English, and living in Montreal. Based on purposeful sampling strategy, a member of the clinical team called eligible participants to obtain initial verbal consent, and then a researcher contacted interested participants, explained the study objectives, and answered questions.

Clinical research coordinators sent clinicians email invitations to participate, explaining the objective of the study. Interested clinicians contacted the researcher *via* email. Clinicians from different professions in rehabilitation, of all years of experience working with individuals with ABI in inpatient, outpatient rehabilitation hospital settings, community care, or delivering home rehabilitation, and who spoke English or French were recruited based on purposeful sampling strategy. All participants signed a consent form before attending the focus group discussions.

### Procedure

Step 1: To facilitate the discussion during the focus group, a description of the purpose of the study was sent to participants ahead of time, along with a demographic questionnaire. Clinicians were also asked a general question to identify mobility measures used in their clinical setting. One week was given to complete the inventory that was compiled across rehabilitation sites and sent to clinician participants.

Step 2: A team of three clinical researchers with expertise in ABI and mobility (RA, SA, CA), reviewed the focus group interview guides and questions for individuals with ABI and clinicians. Iterative changes and reviews of all materials sent to participants were conducted to ensure clarity of the documents. Three focus group discussions with clinicians, one with individuals with stroke, and one with individuals with TBI were conducted between November 2019 and May 2020, and lasted for 90–120 min. A private room was provided for in-person focus groups at all rehabilitation sites except for individuals with TBI, whose focus group was held virtually *via* a web video-conferencing platform (Zoom Video Communications Inc., 2020) due to the COVID19 pandemic. All focus groups were secured using McGill University servers with security protocols. The data from both in-person and virtual focus groups was combined and analyzed as one source [39]. After each focus group, a verbal summary was provided at the end of the discussion to participants to ensure clarity and accuracy of the content.

Two researchers (RA, SA) conducted the focus group discussions with open-ended questions, derived from the study objectives (Table 1). Two co-moderators attended the focus groups and took notes. An observer was present to record non-verbal communication and additional notes. Pseudonyms were assigned to each participant. The audio-recordings complemented the notes and were transcribed verbatim afterwards.

**Table 1.** Focus group questions.

Clinicians guiding questions	
(1)	What are the questions that you have in relation to your practice delivering rehabilitation to individuals with acquired brain injury (ABI)?
(2)	In general, what are the areas that you would like to improve either in your individual practice or rehabilitation among individuals with ABI?
(3)	How do you define mobility? [The focus group members will agree on a definition on mobility that will be read back to the focus group participants (i.e. clinicians)]
(4)	What are the important factors that you believe influence mobility?
(5)	What do you feel you need to evaluate to have a good picture of a person’s mobility while the person with ABI in rehabilitation (inpatient/outpatient)?
(6)	What do you feel you need to evaluate to have a good picture of a person’s mobility while the person with ABI in community?
(7)	From the inventory, how did you choose these measures? Is it capturing all aspects of mobility?
(8)	What is your perception in regards to mobility measures that were captured from the literature and were not proposed in the inventory?
(9)	Consider mobility in rehabilitation setting; what do you see are the challenges of using outcome measures in this environment and what can be done to make it easier to use them?
(10)	Now consider mobility in the community; What do you see are the challenges of using outcome measures in this environment and what can be done to make it easier to use them?
(11)	From your experience regarding the use of mobility measures, how do you use the scores to guide the development of the intervention plan?
Individuals with acquired brain injury guiding questions	
(1)	As an individual with stroke or traumatic brain injury, what are the questions that you have in relation to your condition and to care you received since you have your incidence. (This can include care at the hospital, rehabilitation, or with community care providers including your family doctor)?
(2)	What are the areas that you would like to improve in rehabilitation health care system (if any) to get better care?
(3)	What has been your experience in terms of your daily activities, including work or school, or in participating in social activities with family and friends?
(4)	Were you involved as much as you wanted to be in decisions about your care and treatment?
(5)	How do you define mobility? [The focus group members will discuss a definition of mobility that will be read back to the focus group participants (i.e. individuals with ABI)]
(6)	What are the important factors (e.g. cognition, and environment) that you believe influence mobility?
(7)	As someone lives with stroke or traumatic brain injury, what do you feel needs to be measured or monitored in relation to mobility while someone is in the hospital?
(8)	As someone lives with stroke or traumatic brain injury, what do you feel needs to be measured or monitored in relation to mobility in community?
(9)	Please explain how rehabilitation care prepared you to return home/ back to your work (if relevant)/ school (if relevant), and community?
(10)	Consider mobility in rehabilitation setting; what are the challenges that you face in this environment and what can be done to overcome these challenges?
(11)	Now consider mobility in the community; what are the challenges that you face in this environment and what can be done to overcome these challenges?

### Data analysis

Descriptive statistics summarized the characteristics of participants. As described below, the thematic analysis was based on an inductive thematic content analysis, as described by Creswell [38], and a deductive thematic content analysis using the ten rules for the ICF linking process [40] (Supplementary Appendix 1).

#### Data coding

In the first stage, a short debriefing was completed after each focus group. Notes taken by the co-moderators assisted in the analysis. All focus groups were transcribed verbatim by the first author. The first author familiarized herself with the data by repeatedly reading and listening to the recordings and documenting initial ideas arising from the audio and verbal materials [38].

During the second stage, two independent researchers (RA, DR) read each of the transcripts to gain a sense of the data. Then, line-by-line coding was undertaken independently using an open-ended approach to capture ideas expressed by participants. The process was done by coding terms that were as broad-based as possible to avoid premature closure on interpretation. Handwritten notes from the co-moderators and observer were also consulted. Final codes were established by comparing the codes of both researchers and reviewing the content considering the explicit aims of the study [38]. Disagreements were resolved by discussion and consensus. If not resolved, a third reviewer was consulted.

During the third stage, ten rules established for the ICF linking process [40] were used to analyze the data deductively by the first author and then verified by the second author. The codes of each quote were linked to the ICF domains of Body Function, Activity and Participation and Contextual Factors. Then, linked at a general level (1-level classification) and expanded to levels of greater detail (2nd and 3rd specific ICF category) when the information was available.

A third researcher (SA) independently reviewed the provisional theme summaries from the second and third stage. Through iterative discussion and consultation during a series of virtual meetings, the reviewers verified the themes, and mapped the relationships between them. Reviewers met regularly to resolve any discrepancies, increasing the consistency of the findings.

#### Code rating

In the second stage, the code rating was performed by calculating the frequency of each identified code corresponding to each theme among all participants. This helped to assess saturation based on the level of repetition of codes across all participants [41].

During the third stage, we calculated the proportion of each code in each theme in relation to the ICF domains divided by the total number of codes in the theme. Calculating the proportion of codes within ICF domains helped to explore the prominent ICF domains that mostly identify factors influencing mobility that need to be considered while evaluating mobility among individuals with ABI.

### Triangulation, credibility, and reflexivity

The primary means for ensuring trustworthiness was through triangulation, reflexivity, credibility, and peer debriefing. Conducting a focus group with individuals with ABI to corroborate or contrast with clinician perceptions served as a form of data source triangulation [42]. Meetings between the focus group moderators, co-moderators, and observer to compare notes and to discuss expected and unexpected tangents facilitated reflexivity. Credibility [43] of data collection was established by cross-checking audio-files and transcripts to ensure trustworthiness [44]. Additionally, a verbal summary of the discussions was provided to the focus group participants to ensure the accuracy and credibility of the data. Data analysis involved regular discussions between the reviewers in assessing independently coded data and themes. Having multiple independent researchers code transcripts and compare codes through peer debriefing was a form of researcher triangulation and encouraged reflection on and refinement of categories as they were formulated [42].

## Results

### Participant characteristics

Seventeen clinicians from different professions (physiotherapists, occupational therapists, speech therapists, psychologist, and social worker) agreed to participate in the study (Table 2). Each group included 3 to 10 participants. They had an average of 11.89 ± 7.04 and 10.82 ± 7.05 years of experience working with stroke and TBI population, respectively. The fourth focus group among individuals with stroke included five participants. The majority of the sample was men (80%), and the mean age was 58.4 ± 15.69 years. The severity of the injury for most participants was moderate (60%), as determined using the National Institutes of Health Stroke Scale [45]. The last one was conducted among five female participants with TBI. The mean age was 43 ± 17.24 years, and the severity of injury for most participants was mild (80%). The severity of injury was categorized based on the modified-Glasgow Coma Scale [46]. The demographic information of individuals with ABI is presented in Table 3. The inventories we received from three CRIR rehabilitation sites between August-September 2019 included 49 measures used to evaluate mobility among individuals with ABI (Supplementary Appendix 2).

**Table 2.** Characteristics of clinicians.

Variables	Focus groups (*n* = 3); sample size: (*n* = 17) *n* (%)
Age (years)	
20–39	6 (35)
40–59	11 (65)
Age (*M* ± *SD*) years	41.35 ± 10.28 years
Sex	
Male	1 (6)
Female	16 (94)
Affiliated rehabilitation sites of CRIR	
CRDM	4 (23)/Stroke care
IURDPM	3 (17)/Stroke care
JRH	10 (59)/TBI care
Profession	
Physiotherapists	6 (35)
Occupational therapists	6 (35)
Speech therapists	1 (6)
Psychologist	2 (12)
Social worker	2 (12)
Work position	
Full time/Permanent	13 (76)
Full time/Temporary	1 (6)
Part time/Permanent	2 (12)
Part time/Temporary	1 (6)
Work settings	
Primary care	2 (12)
Secondary care	10 (59)
Tertiary care	5 (29)
Years of work experience (*M* ± *SD*) years	
Practice (in general)	15.79 ± 8
Practice with stroke	11.89 ± 7.04
Practice with TBI	10.82 ± 7.05

ABI: acquired brain injury; CRIR: Centre for Interdisciplinary Research in Rehabilitation of Greater Montreal; CRDM: Constance Lethbridge Rehabilitation Centre; IURDPM: Institut universitaire sur la eadaptation en déficience physique de Montréal; JRH: Jewish Rehabilitation Hospital; TBI: traumatic brain injury.

**Table 3.** Characteristics of individuals with acquired brain injury.

Variables	Individuals with stroke Focus group (*n* = 1); sample size (*n* = 5) *n* (%)	Individuals with TBI Focus group (*n* = 1); sample size (*n* = 5) *n* (%)
Age (years)		
20–39	1 (20)	2 (40)
40–59	2 (40)	3 (60)
60–79	2 (20)	
Age (*M* ± *SD*) years	58.4 ± 15.69	43 ± 17.24
Sex		
Male	4 (80)	5 (100)
Female	1 (20)	
Affiliated rehabilitation sites of CRIR		
CRDM	5 (100)	5 (100)
Education		
Secondary school	2 (40)	
Bachelor degree	3 (60)	5 (100)
Marital status		
Married	3 (60)	2 (40)
Divorced	1 (20)	1 (20)
Single	1 (20)	2 (40)
Employment		
Full time worker	1 (20)	2 (40)
Part time worker		1 (20)
Unemployment	2 (40)	
Retired	2 (40)	2 (40)
Severity of injury		
Mild	2 (40)	4 (80)
Moderate	3 (60)	1 (20)
Severe		
Number of years living with ABI		
≤6 months	1 (20)	1 (20)
6 months–1 year		2 (40)
1–2 years	4 (80)	2 (40)
Number of years (range)	9 months–3 years	6 months–2 years
Type of focus group	Face-to-face	Virtual-conferencing
Type of technology used		
Iphone/ipad	Not applicable	2 (40)
Desktop		2 (40)
Laptop		1 (20)

ABI: acquired brain injury; CRIR: Centre for Interdisciplinary Research in Rehabilitation of Greater Montreal; CRDM: Constance Lethbridge Rehabilitation Centre.

### Emerged themes

The coding rating for each theme by clinicians and individuals with ABI was presented in Supplementary Appendix 3.

### Theme 1: considering mobility holistically and individual needs, preferences, and unique experiences

#### A Comprehensive definition of mobility

It was first necessary to understand how clinicians and individuals with ABI define mobility. Individuals with ABI defined mobility as the ability of the person to walk independently (*n* = 4; 40%). Also, clinicians and individuals with ABI explained that mobility is not only walking, as it is influenced by many factors, such as cognition (clinicians: *n* = 2; 12%; individuals with ABI: *n* = 2; 20%) followed by emotions, such as anxiety (clinicians: *n* = 2; 12%; individuals with ABI: *n* = 1; 10%), fear (clinicians: *n* = 2; 12%), and safety perceptions (clinicians: *n* = 3; 17%).

*C01: “Mobility is a big topic that we deal with; it is not just a physical capacity, all the motivation, cognitive, planning”*

*C05: “The notion of feeling safe of being comfortable with moving versus moving from point A to point B”*

*S05: “I just think mobility, is getting from point A to point B, pretty much”*

*S04: “It was just for me very psychological, that would hinder [mobility]”*

#### Factors hindering mobility, participation, and reintegration into the community

The most common factors limiting mobility identified by individuals with ABI were cognition (*n* = 4; 40%) and fatigue (*n* = 4; 40%) among individuals with stroke; headache (*n* = 4; 40%), fear (*n* = 2; 20%), nausea (*n* = 2; 20%), and dizziness (*n* = 2; 20%) among individuals with TBI. Individuals with ABI explained that some factors, such as cognition lead to a change in self-identity (*n* = 3; 30%). Clinicians reported that cognition (*n* = 1; 6%) and fear of falling (*n* = 1; 6%) resulted in insecurity and limited persons' mobility.

*T05: “It has been almost a year since my concussion symptoms have been lingering. I am confused, have headaches, nausea, double vision, hallucinations, memory problems”*

*S04: “And it gets tiring to [to do your work], but maybe lazy tired, discouraged, depressed, whatever it is”*

*S03: “I remember saying I do not feel like myself”*

*S03: “When something happens to you it affects your family as it did with me”*

*C05: “Fear of falling even if their the balance has improved, they have remained really insecure”*

#### Impacts of bio-psychosocial factors on everyday life and mobility

Participants with ABI discussed that cognitive impairments (*n* = 8; 80%), sensitivity to stimulation (*n* = 4; 40%), comprehension (*n* = 4; 40%), followed by visual (*n* = 2; 20%) and auditory (*n* = 2; 20%) impairments, impacted their ability to participate in daily activities, including social events with family and friends (*n* = 2; 20%), returning to work (*n* = 6; 60%), leisure activities (*n* = 3; 30%) and driving (*n* = 2; 20). All these factors led to self-isolation that impacted their mobility and ability to participate in the community (*n* = 2; 20%).

*T02: “I love my brain. I want it back”*

*T04: “I have too much fear all the time when I am driving”*

*T02: “Going specializing in any kind of sort of taking socializing family gatherings with going to restaurants, cafes you know whether, it was in movies or anything that was loud; all those things were very difficult for me”*

*T02: “I couldn't do the basic work of checking their work and sending emails that I've developed an anxiety and phobia around this and I had to give it up yesterday”*

*T03: “I was already self-isolating because I couldn't handle all the noise”*

### Theme 2: assessment and intervention guidelines

#### Finding common goals with patients

Clinicians explained various assessment methods to evaluate patients’ mobility. Clinicians explained that they tended to integrate the proficiency and judgement they acquired in clinical practice in deciding what tool to use to assess mobility (*n* = 8; 47%). Also, they tended to evaluate mobility among individuals with ABI using alternative methods, such as situational assessment and observations (*n* = 9; 53%) more often than standardized measures.

*C07: “[We assess our patients focusing] more at the level of functional mobility, then more in the community and in using public transportation”*

*C01: “We all use our clinical decision making, our experience to say what would be the most important tool to use”*

Clinicians explained that patient objectives and their clinical judgements (*n* = 8; 47%) were an essential part of the assessment and treatment cycle, highlighting the importance of tailoring rehabilitation to specific deficits and working towards the person’s recovery progress goals. Also, they mentioned that identifying red flags (such as risk of falls) is essential, as these may require additional evaluation (*n* = 2; 12%).

*C03:”Part of the assessment is also establishing the persons' self-reported difficulties, what they perceived to be difficult is a good starting to evaluate”*

*C03: “What are the red flags that require an intervention? Fear, problems with vision, pain, depression, fatigue, dizziness, headaches, if they mention any of these problems it may require other evaluations”*

Some clinicians expressed that using only self-reported questionnaires may not identify disability in patients lacking awareness (*n* = 4; 23%). Also, they reported that self-report measures and screening assessment can only be used on the first contact to highlight individuals' needs (*n* = 10; 59%). They expressed that it is difficult to use self-report questionnaires in cases of aphasia, comprehension or cognitive impairments (*n* = 6; 35%), and it is better to use proxy-reported outcomes (*n* = 1; 6%).

*C03: “We have what the clients subjectively report is their difficulty but we also have a professional responsibility to screen everything that they might not think of”*

*C07: “Using a questionnaire, it's still too much at the beginning if there is a bit of aphasia in there, comprehension problem, are not able to read, or you know they are able to just say simple answers”*

Clinicians reported that standardized measures could be used when assessing patients to point out their impairment and limitation levels and to be able to track changes during follow-up sessions (*n* = 2; 12%). They highlighted the importance of using both standardized measures and clinical judgement when assessing mobility (*n* = 11; 65%). A clinician reported the importance of using standardized measures when a situational assessment alone cannot give the full picture of a patient's impairment. Another clinician suggested the importance of consistency in measures used across the continuum of care to enable comparison between patients and to track individual progress.

*C04: “Clinical judgment and the degree of the sensitivity to change to target functional abilities in the community”*

*C02: “If the inpatient and the acute outpatient and the chronic outpatient all use the same test, then we can track measures across the time”*

*C02: “I would say that the only time I go with score it is for driving because I cannot go and evaluate a driving by a mise en situation [i.e. situational]”*

Also, a clinician highlighted that standardized measures are becoming more practical to support clinician's recommendations, tracking changes, and discharge planning, but not to establish an intervention plan.

*C01: “I use the objective tool as an argument to support my recommendation”*

Furthermore, clinicians in rehabilitation acknowledge the importance of information exchange, as interdisciplinary collaborative decision-making facilitated aligning treatment planning with patients’ needs (*n* = 10; 59%).

*C07: “The intervention plan depends if it's the disciplinary or interdisciplinary plan”*

#### Challenges clinicians face when they evaluate mobility

Clinicians expressed challenges with using standardized measures (*n* = 9; 32%), as some of them take up an entire patient evaluation session, leaving no time for delivering treatment or education. Other measures can be fatiguing for individuals, which may affect the assessment and treatment cycle. They also reported that some standardized measures are not adapted for use in the community. Clinicians explained that limited tools use guidelines make evaluating mobility more challenging (*n* = 2; 12%). Thus, a shortlist of the most important mobility measures covering different domains is needed.

*C04: “BBS [Berg Balance Scale] is really good but it took 30 minutes. The BESTest [Balance Evaluation Systems Test] took 45 minutes to finish, I mean there are too many things to look at instead of using a tool”*

*C03: “Fatigue is another obstacle if you have to do the Borg [Borg Rating of Perceived Exertion]over three visits”*

*C05: “Sometimes in the community, it's hard to use a standardize measure to evaluate mobility because of a different environment, so it is more functional”*

*C03: “What we need to know and you know in terms of research questions, what are the top 5, top 10 tests that are going to be helpful”*

A clinician reported the importance of using a practice style that allows the patient to trust the clinician's guidance while also being involved in their care plan, to the extent that the patient wishes to be involved. Other barriers to assessment reported by clinicians were related to environmental factors, such as winter weather (*n* = 2; 12%), as persons with ABI tend to isolate themselves at home. This may increase the difficulties in using assistive devices. Also, alcohol and drug abuse (*n* = 2; 12%) may result in falls and harms assessment and treatment sequences that impact mobility negatively.

*C01: “Another barrier for sure is the client themselves in term of fear, do they trust you, or even if they trust you are they able to put themselves in a situation where they are challenged”*

*C01:”[Clients were] homebound in winter because either they don't have the confidence or just very difficult to get out in a wheelchair, probably a combination of the two?”*

*C05: “We have clients with a problem of abusive consumption when they return home and resume their consumption, they will have falls”*

Clinicians reported that safety issues (*n* = 8; 47%), cognitive impairment (*n* = 7; 41%), and patients’ confidence (*n* = 3; 17%) were significant barriers to assessment. They explained that some individuals with ABI overestimated their abilities and showed a lack of fear, awareness, and judgement that impacted their safety when they reintegrated into the community.

*C07: “When we talk about cognitive versus physical, it depends on the clients, there are clients for whom the cognitive dominates, which make them unsafe to cross the street, they don't orient themselves in their neighbourhood”*

#### Engaging the patient and considering their perspective in their care

Individuals with ABI, specifically stroke, explained that using an engaging communication style can help them feel comfortable and involved in the care process (*n* = 7; 70%). They usually ask questions regarding the purpose of the evaluation and treatment provided to understand their benefits. On the other hand, some participants with stroke tend not to ask questions related to the evaluation or the treatment provided to them (*n* = 2; 20%). They reported that healthcare providers know exactly what to do as they follow a strict protocol for evaluation and treatments. One participant with stroke reported that he learned how to say “no” for certain evaluations and treatments because he thought it was a bad decision made by healthcare providers.

*S02: “I would actually stop at the beginning and ask what do you want to gain out of this, like what's the purpose of it”*

*S05: “I learned there [i.e. in hospital][how] to say no to certain things because they would really bad decision [taken by the clinicians]”*

### Theme 3: support network

The theme of support network described a number of influential factors around an individual (e.g. family) sharing responsibility with individuals with ABI to influence mobility and help them reintegrate better into the community.

#### Caregiver support

Clinicians highlighted the importance of caregiver support (*n* = 5; 29%) as a secondary source of care for individuals with ABI, especially if they have cognitive impairments, to facilitate their mobility and provide the essential support to discuss their limitations. Communication was often hindered by temporary or permanent impairment. Therefore, a family member is needed to communicate with clinicians on behalf of people with such impairments.

*C06: “A family member or a caregiver can help especially for patients with cognitive issues”*

One of the adjustments to new life roles that clinicians perceived as important to improve the self-identity and coping skills of individuals with ABI was to have support from their family members, especially when they are new to using assistive devices (e.g. wheelchair). Also, individuals with ABI reported the importance of having support from a family member, as they can make a positive adjustment to their life (*n* = 8; 80%).

*C01: “A lot of people maybe it is a new thing that their loved one is using a wheelchair”*

*T04: “I think the psychologist and the support from your family is more effective”*

#### Providers support

Participants with ABI acknowledged the support and services provided by some healthcare providers in rehabilitation centres (*n* = 3; 30%). They explained the importance of the healthcare professional listening to the patient’s complaints and understanding what the patient needs, which is not always the case among healthcare professionals.

*T02: “I thought the team of the [rehabilitation] was very good. They were on board, I felt finally really supported”*

A social worker explained that they offer support to families who have loved ones with a disability to develop coping strategies to help them understand the patient’s impairment and how they can assist them in integrating into daily activities within the community.

*C13: “We work with the families, so it is important to get their point of view and their input and to help them to cope into the situation to help the patient”*

#### Community support

Individuals with ABI explained that community support by the public and the availability of governmental resources to meet the needs of persons with disabilities is needed (*n* = 4; 40%). Participants with ABI reported how the perceptions of people at some institutions *vs.* community differed, and how stigma associated with ABI impacts mobility (*n* = 2; 20%).

*S06: “I find one thing quite annoying is that when you are put around in a wheelchair, people look at you, and some taxi drivers, they think your brain is gone, they think you stupid”*

Clinicians explained the importance of supporting persons with disabilities to facilitate their mobility, but because of limited community resources, clinicians cannot provide the needed support (*n* = 4; 23%). There is a need to determine the best way to provide community support through guidelines and policy services that are limited. Also, clinicians discussed that support services are especially lacking in the community for persons who live alone.

*C05: “[The support services are missing, especially when the patient] is not [obtaining] the necessary balance, the necessary endurance or because it is not well oriented and safe to cross the street”*

*C06: “They end up after that feeling like there's no one left, there are no services that can be provided for them”*

### Theme 4: uncertainty about symptoms and recovery

Participants with TBI described experiencing confusion and uncertainty about their symptoms and diagnosis when their own experiences did not make sense to them or match what they were being told by the healthcare providers. The uncertainty left them feeling increased distress and impacted their participation in the community (*n* = 3; 30%). Also, they reported that there was uncertainty regarding expected recovery and consequences associated with their injury. Furthermore, participants experienced uncertainty about whether they were still recovering, how to tell if they were getting better, why it was taking so long, and how recovery could be hastened, so they could reintegrate into the community (*n* = 4; 40%).

*T05: “I ask myself if I would ever return normal and would my symptoms last for a lifetime. They recently told me to at xxx that my physiotherapy sessions have ended”*

*T05: “I ask myself if I would ever return normal and would my symptoms last for a lifetime. They recently told me to at xxx that my physiotherapy sessions have ended”*

Moreover, most participants with TBI described the benefits of having constructive strategies to manage their symptoms, but some appeared less confident in their strategies and more concerned about making their symptoms worse by “getting it wrong” (*n* = 4; 40%).

*T03: “I simply write things down, like using notes in my phone or just like a notepad. So I can remind myself, but sometimes I forgot”*

### The ICF linking process

Codes for each theme were mapped to the ICF domains as follows: Theme 1: Body Function (*n* = 46; 54%), followed by Activity and Participation (*n* = 30; 35%), Environmental Factors (*n* = 7; 8%), and Personal Factors (*n* = 2; 2%); Theme 2: Environmental Factors (*n* = 74; 69%), followed by Body Function (*n* = 21; 19%), and Activity and Participation (*n* = 12; 11%); Theme 3: Environmental Factors (*n* = 20; 77%), followed by Body Function (*n* = 6; 23%); Theme 4: Body Function (*n* = 4; 21%), and not covered health condition (*n* = 15; 79%) (Supplementary Appendix 1).

## Discussion

Individuals with ABI and clinicians perspectives yielded an understanding of the factors influencing mobility that need to be considered while evaluating mobility among individuals with ABI. Participants mainly focussed on challenges that limit mobility and provided suggestions of how to address these to incorporate into individualized care management plans to improve mobility while considering individuals with ABI needs and preferences. Through an inductive thematic analysis, four main themes emerged: considering mobility holistically and individual needs, preferences, and unique experiences; assessment and intervention guidelines; support network; and uncertainty about symptoms and recovery. To our knowledge, this is the first time that the ten-rule ICF linking process [40] was used in a deductive thematic analysis to explore the prominent ICF domains that mostly identify factors influencing mobility that need to be considered while evaluating mobility among individuals with ABI.

A combined inductive and deductive thematic analysis was chosen by the authors to best address the research questions. While inductive thematic analysis searched for patterns from raw data, deductive thematic analysis addressed the set of information and searched for consistencies and anomalies [47]. Combining inductive and deductive thematic analysis approaches allowed for a complete analysis and a critical realism ontological approach [47]. While the inductive thematic analysis allowed the reality of others to be clearly represented, the deductive thematic analysis provided an initial grounding of using a common language based on the ICF framework.

### Theme 1: considering mobility holistically and individual needs, preferences, and unique experiences

Our study evaluated the emphasis on factors influencing mobility among individuals with ABI which was not explored in earlier published studies. While discussing factors influencing mobility, most post-stroke survivors mentioned cognition and fatigue, whereas post-TBI survivors mentioned headache, fear, nausea, and dizziness. Clinicians were mostly concerned with individuals’ safety and wanted to prevent falls. Also, we did explore clinicians and individuals with ABI perspectives on factors influencing mobility across the continuum of care to better understand how mobility needs to be evaluated over time. Previous studies did not evaluate mobility comprehensively and focussed mainly on evaluating the perspectives of individuals with stroke about mobility in the context of walking and falling following inpatient rehabilitation or skilled nursing facilities [48–50].

Participants with ABI stated that cognitive impairments and sensitivity to stimulation have a considerable impact on their daily activities, resulting in developing psychological and emotional factors that would lead to self-isolation. Individuals with ABI experience a process of reconstitution of self in response to the burden of living with a deficit or disability. Studies have shown that individuals with chronic conditions tend to actively engage in daily life routines by reflecting on their deficit or disability, which helps them make sense of who they are, experiencing self in a new conscious way [51]. Restoring a sense of control and self-identity is essential for persons with ABI to be able to move and integrate into their community.

Individuals with ABI identified their needs for encouragement and feedback from healthcare professionals, to facilitate their mobility, increase their understanding, and progress to goals within a rehabilitation setting. Fulfilling these needs would increase patients' ability to learn, improve their level of achievement, and underpin their motivation [52]. Several studies included interpretations of data from participants discussing feelings of anxiety and depression during the rehabilitation process [53,54]. Not engaging patients as whole persons and respecting their needs and preferences may lead to a perceived lack of control on the patient’s part, ultimately resulting in feelings of futility, decrease in confidence, and self-isolation [55].

### Theme 2: assessment and intervention guidelines

Our findings revealed that the interchangeable common goals between clinicians and patients can help establish shared goals and priorities to evaluate mobility comprehensively. In evaluating mobility, some clinicians rely on clinical experience and judgement, while others rely on situational assessment and observations. Inventories identified 49 measures that clinicians used to evaluate mobility among individuals with ABI. Clinicians assessed factors that influence mobility (such as cognition). Overall, clinicians appear to regard measurement of mobility in ABI survivors as necessary, but acknowledged the complexity and challenges associated with measuring community mobility in ABI survivors. One challenge identified by participating clinicians was the lack of specific tools for measuring mobility, compelling clinicians to rely on a range of measures that infer mobility, such as tools to assess balance [56] and walking [57,58]. Even then, clinicians were not consistent in which measures to use.

Clinicians also tend to believe that patients usually focus on ultimate outcomes and not the specific deficits or limitations that need to be considered while evaluating mobility. Clinicians identified the importance of adapting assessments and their decisions to the deficits to help patients integrate into the community safely. It might be essential to educate ABI survivors and caregivers to know their deficits and limitations to promote and facilitate information exchange. Clinicians rarely use self-reported questionnaires, as they require considerable time and effort especially if the individuals with ABI are cognitively impaired. It may be appropriate to use alternative methods (such as proxy-reported outcomes) to evaluate an individual’s mobility; ensuring that there is supporting evidence for the measure used to be proxy-reported.

The results supported that individuals with ABI prefer to be actively involved in the rehabilitation process, instead of allowing clinicians to make judgments and decisions on their behalf based only on functional assessments. Previous studies showed that shared decision making between patients and clinicians impacts engagement in rehabilitation [59–61]. Clinicians tend to integrate their proficiency and judgement through clinical practice in deciding which tool to use to assess mobility. Clinicians need to understand the reasoning behind patient preferences to tailor the needed treatment [62]. They should be encouraged to explore how treatment preference matches patient goals, as well as the individuals’ understanding of associated pros and cons. Treatment preferences adapted to patients’ goals should be seen as a process of shared decision making. Patients and clinicians are expected to collaborate and make decisions together that are informed by the best available evidence and genuinely aligned with patient preferences [50]. Thus, healthcare professionals must consider involving patients during all stages of rehabilitation care.

### Theme 3: support network

The disconnect between the expectations of clinicians and ABI survivors can be linked to patient's characteristics, availability of support, social determinants, and health system factors adapted towards discharging patients sooner from the hospital [50]. One way to address this problem is by engaging patients from the outset in the selection of outcome measures and linking evaluations to a care plan that they develop together. In the absence of a support network, patients may be less likely to participate when they feel their emotional needs are not considered, resulting in a decreased sense of self-perception in conjunction with a decreased sense of belonging [51,63]. A patient-centred response to emotions requires reacting to emotional cues [64,65]. Thus, healthcare providers should communicate their understanding of an emotional response and express acknowledgement by showing sympathy, empathy, and reassurance.

### Theme 4: uncertainty about symptoms and recovery

Healthcare professionals need to openly acknowledge, support, and express commitment to the continuity of their patient’s care and provide extra attention to the way social, cultural, psychological and other factors impact a patient’s ability to be involved in their care [66]. The most significant concern for participants was the uncertainty they faced throughout the social distancing and isolation measures during the COVID-19 pandemic, as well as their ability to cope longer-term. There was also uncertainty as to how they would act, with some fear of lingering anxiety over social contact and health, and others eager to return to normal levels of social activity. Another critical component of responding to emotional needs is managing uncertainty among individuals with ABI. It is essential to recognize that sharing information is a value, a behaviour and a skill that may vary depending on a patient's perspective [64]. Sustaining trust between patient and clinician has both instrumental and intrinsic value, as it leads to better patient outcomes while improving the therapeutic experience for both of them [64]. For example, some patients lose trust when uncertain information was given [67]. Thus, providing a patient-centred exchange of information requires sensitivity to the goals and expectations of the patient.

### Common language for measuring mobility

Evaluation of the effectiveness of rehabilitation interventions after ABI is a high priority for clinicians and individuals with ABI [59–61]. However, selection of a suitable outcome measure can pose a challenge to both researchers and clinicians, as the range of outcome measures available in the clinical research literature is vast, and distinctions between them are often not clear. Indeed, numerous studies focussing on mobility outcome measures have been published, many studies highlighting the need for standardized definitions and higher consensus and guidance in outcome selection [56–58]. Researchers and clinicians need to consider the content of measures and whether the domains evaluated match research and clinical objectives.

The use of more comprehensive models that can locate mobility within a framework to identify all the relevant outcomes and the linkage between them and the relationship between them is essential. The ICF is a universally accepted framework used to foster the inclusion of the critical domains which impact an individual with ABI. From our identified themes, it is clinically useful when the stroke and TBI published core sets [68,69] are used to describe mobility domains measured by standardized measures to inform the measures best suited to a holistic approach to care, linking impairments, activity limitations, and participation restrictions. Equal emphasis should be placed on determining the influence of personal and environmental elements on a person's overall health and well-being [24]. This allows the development of an inclusive treatment plan for the individual with ABI where the functional profile is fully considered. An example of improving standardization of outcomes across several research areas is the Core Outcome Measures in Effectiveness Trials (COMET) initiative, which aims to improve development and application of agreed-upon standardized sets of outcomes, the “Core Outcome Sets” [70]. Thus, a future step of our work is to develop a core outcome set of mobility to standardize measures used across clinical sites and studies among individuals with ABI.

### Limitations

Findings of this study are based on a purposive sample and therefore may not represent views of a broader population of clinicians working in a different setting, specifically in the community. Since most of the participants with ABI in the same focus group were recruited from one rehabilitation site, we were unable to reach saturation in the findings between them. It is also possible that participants might not have mentioned all the factors that influence mobility because of the open-ended discussions. Hence, the results of this study should be interpreted cautiously. Future researchers may further distinguish the impact on caregiver experiences along the care continuum, contributing to the provision of timely support to improve health outcomes.

Conducting focus group discussions online has become a popular method for collecting qualitative data. Advances in technology have enabled researchers to adapt in-person focus group methods for use in an online environment [39,71]. Although there is a great deal of interest in online focus group methods, less attention has been given to the quality of data they generate in comparison with the in-person focus group. In comparison to the in-person focus group, the virtual one allowed participants to take part from a familiar environment instead of meeting in the same space [71]. This may reduce costs for both researchers and participants, such as the unnecessary need to travel. The results suggest that the role of the moderator in either setting could influence the data that was generated [39]. In the in-person focus group, not every participant was able to speak due to time constraints and some participants dominating the conversation. In the virtual one, nearly all the participants were able to express their opinions. Moderators in an in-person focus group must work harder to control the flow of the discussion. Questioning, however, proved to be more difficult in the virtual focus group, as non-verbal or visual cues were harder to observe to allow the moderator to clarify further discussions [39,71]. Although it is difficult to determine whether the differences occurred as a result of the focus group type, the findings suggest that the themes obtained from both formats were similar despite variations in word count per response.

## Conclusion

This study has presented clinicians and individuals with ABI perspective of factors influencing mobility that need to be considered while evaluating mobility; and to incorporate individuals with ABI needs and preferences into individualized care management plans among individuals with ABI. Comprehensive measurement of mobility remains an ongoing challenge owing to multiple contributing factors, ranging from personal and psychosocial factors to the effect of myriad environmental community situations. This study suggests a need to raise awareness about engaging patients in their care, and respecting their needs and preferences. Healthcare professionals should provide the needed communicative tools to their patients to improve patient-centred care.

## Supplementary Material

Supplemental Material

## Data Availability

Data available within the article.
