# Supplementary material for: Clinicians and individuals with acquired brain injury perspectives about factors that influence mobility: creating a core set of mobility domains among individuals with acquired brain injury
Source: Ann Med. 2021 Dec 13;53(1):2365–79. doi: 10.1080/07853890.2021.2015539 (PMC8676689; doi:10.1080/07853890.2021.2015539)
Supplement: Supplemental Material [file IANN_A_2015539_SM7437.zip › Supplemental files/Appendix 1.docx]

**Appendix 1. Inductive and deductive thematic analysis**

| **Quotes** | **Population** | **Formulated statements** | **Codes** | **ICF component** | **1st Level ICF category** | **2nd Level ICF category** | **3rd or higher Level ICF category** |
| --- | --- | --- | --- | --- | --- | --- | --- |
| **Theme 1. Considering mobility holistically and individual needs, preferences and unique experiences** | | | | | | | |
| **1.1. A comprehensive definition of mobility** | | | | | | | |
| *C01:“mobility is a big topic that we deal with; it is not just a physical capacity, all the motivation, cognitive, planning, that can go just for that one appointment; need to be taken into account”* | clinician | Bio-psycho-social modal as a lens/frame to view mobility and what impacts it | Physical capacity Cognition Planning Motivation | Activity and Participation Body Function | d4 Mobility  b1 Mental Function | d499 Mobility, unspecified  b144 Memory functions  b164 Higher-level cognitive functions  b130 Energy and drive functions | b1449 Memory functions, unspecified  b1641 Organization and planning  b1301 Motivation |
| *C01: “travel, work and for leisure activities, biking is relate to mobility”* | clinician | Mobility: travel; work, leisure | Recreation Work  Leisure activities  Biking | Activity and Participation | d9 Community, social and civic life d8 Major life areas | d920 Recreation and leisure Work and employment (d840-d859)  d920 Recreation and leisure | d9209 Recreation and leisure, unspecified d8509 Remunerative employment, unspecified  d9209 Recreation and leisure, unspecified  d9201 Sports |
| *C05:“safety because sometimes things are going well with our patients, but they're still very insecure”* | clinician | Mobility definition: add safety | Safety | Environmental Factor | e5 Services, systems and policies | e530 Utilities services, systems and policies | e5308 Utilities services, systems and policies, other specified (Safety standards for individuals with ABI) |
| *C05:“the notion of feeling safe of being comfortable with moving versus moving from point A to point B, but with a very high level of anxiety, they don't want to do it anymore”* | clinician | Mobility definition: add safety and fear, anxiety | Safety  Walking Fear Anxiety | Environmental Factor  Activity and Participation  Body Function | e5 Services, systems and policies  d4 Mobility  b1 Mental Function | e530 Utilities services, systems and policies  Walking and moving (d450-d469)  b152 Emotional functions | e5308 Utilities services, systems and policies, other specified (Safety standards for individuals with ABI)  b1528 Emotional functions, other specified (anxiety)  b1522 Range of emotions |
| *C05: "we can add self confidence, fear and anxiety as factors that influence mobility"* | clinician | Mobility definition: add safety and fear | Safety Fear Self-confidence Anxiety | Environmental Factor  Body Function | e5 Services, systems and policies  b1 Mental Function | e530 Utilities services, systems and policies  b152 Emotional functions  b126 Temperament and personality functions | e5308 Utilities services, systems and policies, other specified (Safety standards for individuals with ABI)  b1266 Confidence  b1528 Emotional functions, other specified (anxiety)  b1522 Range of emotions |
| *C01: “self-efficacy is predictor for re-integration into the work place, into society”* | clinician | Mobility: self efficacy, community | Self-efficacy | Body Function | b1 Mental Function | b180 Experience of self and time functions | b1800 Experience of self |
| *C02: “defiantly, re-integration is a factor that influence mobility”* | clinician | Mobility: community re-integration | Community re-integration | Activity and Participation | d9 Community, social and civic life | d999 Community, social and civic life, unspecified |  |
| *C11: “So If you really trying to put the finger on mobility in TBI, the big part of it is a cognitive issue and the behaviour”* | clinician | Cognitive deficits and behaviour impact on mobility | Cognitive deficits Behaviour | Body Function | b1 Mental Function | b144 Memory functions b164 Higher-level cognitive functions b147 Psychomotor functions | b1449 Memory functions, unspecified b1649 Higher-level cognitive functions, unspecified b1480 Psychomotor control |
| *S01: “I think, the first thing I can think of is mobility is just ambulating, walking, that's what normally people would say, you know. They don't think about, maybe the bigger picture”* | stroke | Mobility definition: mobility is walking, but it is also much more | Walking | Activity and Participation | d4 Mobility | Walking and moving (d450-d469) |  |
| *S03: “Walking, driving, whatever, I think it is a very physical thing”* | stroke | Mobility definition: walking, driving, a physical thing | Walking Driving | Activity and Participation | d4 Mobility | Walking and moving (d450-d469) d475 Driving | d4759 Driving, unspecified |
| *S02: “So, mobility, and it's kind of stole a lot from my independence, you know”* | stroke | Mobility definition: what is lost post-stroke | Self-identity | Body Function | b1 Mental Function | b180 Experience of self and time functions | b1800 Experience of self |
| *S05: “I just think mobility, is getting from point A to point B, pretty much, Yeah I do it, I don't enjoy it, but I get around”* | stroke | Mobility definition: getting from one point to another | Walking | Activity and Participation | d4 Mobility | Walking and moving (d450-d469) |  |
| *S04: “it was just for me very psychological, that would hinder and then, I wasn't travelling, I would not go anywhere”* | stroke | Fear, psychological factors impact on mobility; change in identity | Psychological  Travel | Body Function  Activity and Participation | b1 Mental Function  d4 Mobility | b199 Mental functions, unspecified  Moving around using transportation (d470-d489) |  |
| *T05: "Where I'm getting messed something up. So even taking the bus to rehab, you know, am I going to get off at the right stop. Can I remember the street?"* | TBI | Memory impacts on mobility; routines, maps | Memory | Body Function | b1 Mental Function | b144 Memory Functions |  |
| *T04:" So they [people] really like it's hard to navigate your social life, your family life, your work life like, all of it like I have gone"* | TBI | Navigating recovery with family, work and social life is difficult. All domains of life are challenging post-TBI | Cognition Work Social life Relationship with family | Body Function  Activity and Participation | b1 Mental Functions  d9 Community, social and civic life d7 Interpersonal interactions and relationships d8 Major life areas | b144 Memory functions  b164 Higher-level cognitive functions  d910 Community life d760 Family relationships Work and employment (d840-d859) | b1449 Memory functions, unspecified b1649 Higher-level cognitive functions, unspecified  d7609 Family relationships, unspecified |
| *T02:"Now, I haven't received any money compensation from xxx to which has been a problem and which contributed to anxiety, which I developed turned into self-isolation"* | TBI | Financial difficulty; lack of services impact on mobility; anxiety | Finances Anxiety Self-isolation | Environmental Factors  Body Function | e1 Products and Technology  b1 Mental Function | e165 Assets  b152 Emotional functions b180 Experience of self and time functions | e 1650 Financial assets  b1528 Emotional functions, other specified (anxiety) b1800 Experience of self |
| **1.2. Factors hindering mobility, participation and reintegration into the community** | | | | | | | |
| *S02: “When I am tired, I still screw my words”* | stroke | Factors: fatigue impact on word finding | Cognition Fatigue Finding words/Speech | Body Function | b1 Mental Functions  b4 Functions of the cardiovascular, haematological, immunological and respiratory systems b3 Voice and speech functions | b144 Memory functions b455 Exercise tolerance functions  b330 Fluency and rhythm of speech functions | b4552 Fatigability  b3309 Fluency and rhythm of speech functions, unspecified |
| *S04:“And it gets tiring to [to do your work], but maybe lazy tired, discouraged, depressed, whatever it is”* | stroke | Fatigue, and other deficits lead to depression and feedback loop limiting mobility and participation | Fatigue Depression | Body Function | b4 Functions of the cardiovascular, haematological, immunological and respiratory systems b1 Mental Function | b455 Exercise tolerance functions  b152 Emotional functions | b4552 Fatigability  b1528 Emotional functions, other specified (depression) |
| *S03 “I was so vulnerable at that point, I mean emotionally, physically”* | stroke | Emotion and vulnerability impact mobility and participation | Emotional  Physical | Body Function  Activity and Participation | b1 Mental Functions  d4 Mobility | b152 Emotional functions  Walking and moving (d450-d469) Moving around using transportation (d470-d489) | b1529 Emotional functions, unspecified |
| *S02: “I don't get around the city anymore, I tried it a bit of walking you know, I do some walking, but I find it's like it's just fatigue”* | stroke | Fatigue impacts on walking; physical activities and mobility | Fatigue | Body Functions | b4 Functions of the cardiovascular, haematological, immunological and respiratory systems | b455 Exercise tolerance functions | b4552 Fatigability |
| *S04: “it is only the one task, and if I picked the wrong one task you can do or schedule for the week involves, going here, going there. I know by this night here, I'm a veggie”* | stroke | Fatigue, energy levels limiting mobility and participation; setting a routine for morning and afternoon can be helpful | Fatigue | Body Functions | b4 Functions of the cardiovascular, haematological, immunological and respiratory systems | b455 Exercise tolerance functions | b4552 Fatigability |
| *S04: “In my case it is the memory thing”* | stroke | Memory and other cognitive issues impact on mobility | Memory | Body Function | b1 Mental Functions | b144 Memory functions |  |
| *S03: “Me it was psychological, what was hindering my mobility just being petrified to leave the house, and what if it happens again and what if I was alone”* | stroke | Fear, psychological factors impact on mobility | Fear Psychological | Body Function | b1 Mental Function | b152 Emotional Function b199 Mental Functions, unspecified | b1522 Range of emotion |
| *S03: “I remember saying I don't feel like myself”* | stroke | Change in identity post stroke; impact on mobility | Self-identity | Body Function | b1 Mental Functions | b180 Experience of self and time functions | b1800 Experience of self |
| *S02: “It's like giving up a life, that you knew and you get in a new life”* | stroke | Change in identity post stroke; impact on mobility | Self-identity | Body Function | b1 Mental Functions | b180 Experience of self and time functions | b1800 Experience of self |
| *S03: “I didn't experience, I mean other than, I lost my license, but I wasn't allowed to drive for a few months after my stroke”* | stroke | Loss of driver's license post-stroke | Loss of driving license Independence Self-identity  Fear | Activity and Participation  Body Function | d4 Mobility  d2 General tasks and demands  b1 Mental Functions | d475 Driving  d210 Undertaking a single task  d220 Undertaking multiple tasks  b180 Experience of self and time functions  b152 Emotional Function | d2109 Undertaking single tasks, unspecified  d2209 Undertaking multiple tasks, unspecified  b1800 Experience of self  b1522 Range of emotion |
| *S03: “when something happens to you it affects your family as it did with me, and my husband and my kids”* | stroke | consequence of the deficits: affect family member (emotionally) | Emotion Relationship with family | Body Function  Activity and Participation | b1 Mental Functions  d7 Interpersonal interactions and relationships | b152 Emotional functions d760 Family relationships | b1529 Emotional functions, unspecified d7608 Family relationships, other specified (husband and kids) |
| *T05: “It's been almost a year since my concussion symptoms have been lingering. I am confused, have headaches, nausea, double vision, hallucinations, memory problems, and am still a far way from making progress”* | TBI | Deficits; nausea, headaches, vision, memory; difficult to make progress and recover | Confusion Memory Double vision Nausea Headache Hallucination  Dizziness | Body Function | b1 Mental Functions  b2 Sensory functions and pain | b199 Mental functions, unspecified  b156 Perceptual functions  b210 Seeing functions  b240 Sensations associated with hearing and vestibular function  b298 Sensory functions and pain, other specified (headache) | b1565 Visuospatial perception  b2109 Seeing functions, unspecified  b2403 Nausea associated with dizziness or vertigo  b2401 Dizziness |
| *T04:“after a brain injury, I lost my sleeping habit”* | TBI | Change in sleeping routines | Sleep disturbance | Body Function | b1 Mental Functions | b134 Sleep functions | b1343 Quality of sleep |
| *T02: "You think you can pull out of it, but you just can't [i.e. symptoms], it is so like am I dreaming this nausea, hangover, headache, be dizzy, unable to focus if I just put my mind to"* | TBI | Cognitive and physiological deficits impact on mobility | Cognitive Nausea Dizziness Headache | Body Function | b1 Mental Functions  b2 Sensory functions and pain | b199 Mental functions, unspecified  b240 Sensations associated with hearing and vestibular function  b298 Sensory functions and pain, other specified (headache) | b2403 Nausea associated with dizziness or vertigo  b2401 Dizziness |
| *T02:"I'm going to say the most disruptive immediate one, of course, is the headaches";* | TBI | Headaches disruptive to participation in life | Headaches | Body Function | b2 Sensory functions and pain | b298 Sensory functions and pain, other specified (headache) |  |
| *T02:" they don't respond to a Tylenol or Advil. They come with a course for hours or a day. They completely put me out of there to be disruptive and can actually put me out of focus”* | TBI | Headaches disruptive to participation in life | Headaches | Body Function | b2 Sensory functions and pain | b298 Sensory functions and pain, other specified (headache) |  |
| *C05: “fear of falling even if their the balance has improved, they have remained really insecure"* | clinician | Mobility factors: safety add fear | Safety Fear | Environmental Factor  Body Function | e5 Services, systems and policies  b1 Mental Functions | e530 Utilities services, systems and policies  b153 Emotional Functions | e5308 Utilities services, systems and policies, other specified (Safety standards for individuals with ABI)  b1522 Range of emotion |
| *C04: “so they feel like they are dizzy and after awhile they start losing confidence in them, so they think that they will fall but they don’t because they can regain their balance, they don’t have balance issue, it is just because that their brain has difficulty”* | clinician | Mobility factors: dizziness, loss confidence, fall, balance, cognition | Dizziness Confidence Balance Cognition Fall | Body Function  Activity and Participation | b2 Sensory functions and pain  b1 Mental Function  d4 Mobility | b240 Sensations associated with hearing and vestibular function  b126 Temperament and personality functions b144 Memory functions b164 Higher-level cognitive functions  Changing and maintaining body position (d410-d429) | b2401 Dizziness  b1266 Confidence  b1449 Memory functions, unspecified  b1649 Higher-level cognitive functions, unspecified |
| **1.3. Impacts of biopsychosocial factors on everyday life** | | | | | | | |
| *T04:“I have too much fear all the time when I am driving”* | TBI | Fear of driving impact mobility | Fear  Driving | Body Function  Activity and Participation | b1 Mental Functions  d4 Mobility | b152 Emotional functions  d475 Driving | b1522 Range of emotion  d4759 Driving, unspecified |
| *T02: “This is just the psychological stuff has been really hard”, “the psychological stuff nipping in my life and not being outside of my life”* | TBI | Psychological impact of TBI on mobility | Psychological Social life | Body Function  Activity and Participation | b1 Mental Functions  d9 Community, social and civic life | b199 Mental functions, unspecified  d910 Community life | d9109 Community life, unspecified |
| *T03:“It’s hard to explain to people that, you know, Oh, I can't remember your name. Lots of your memories gone, your ability to focus on anything is gone"* | TBI | Memory, attention, executive functioning deficits impact on mobility | Cognitive  Social life | Body Function  Activity and Participation | b1 Mental Functions  d9 Community, social and civic life | b164 Higher-level cognitive functions  b199 Mental functions, unspecified  d910 Community life | d9109 Community life, unspecified |
| *T02: "I couldn't go through and read all of their work. I have the same issue of the ability to at some point, my eye, brain gets tired and I can't actually focus"* | TBI | Cognitive issues impact on return to work and mobility | Cognitive  Reading/Comprehension Work | Body Function  Activity and Participation | b1 Mental Functions  d1 Learning and applying knowledge  d8 Major life areas | b199 Mental functions, unspecified b164 Higher-level cognitive functions  Applying knowledge (d160-d179)  Work and employment (d840-d859) |  |
| *T02: "There’s no longer the ability to actually make out words. So then I know I'm tired. My brain is tired and I have to stop [working]"* | TBI | Cognitive, reading, comprehension, and fatigue impact on return to work | Cognitive  Fatigue Reading/Comprehension Work | Body Function  Activity and Participation | b1 Mental Functions  b4 Functions of the cardiovascular, haematological, immunological and respiratory systems  d1 Learning and applying knowledge  d8 Major life areas | b199 Mental functions, unspecified  b164 Higher-level cognitive functions  b455 Exercise tolerance functions  Applying knowledge (d160-d179)  Work and employment (d840-d859) | b4552 Fatigability |
| *T02: "But it's the cognitive stuff, which is that I might read something three times and I still don't actually catch the meaning"* | TBI | Cognitive issues (reading, comprehension) impact on mobility | Cognitive  Reading/Comprehension Work | Body Function  Activity and Participation | b1 Mental Functions  d1 Learning and applying knowledge  d8 Major life areas | b199 Mental functions, unspecified b164 Higher-level cognitive functions  Applying knowledge (d160-d179)  Work and employment (d840-d859) |  |
| *T01: "There were days [at work station] where I was just like, it's like you're okay. And all of a sudden you're kind of just like you'd walk in somewhere and you're like, I don't really remember what I came in here for"* | TBI | Memory impacted while navigating from place to place, not remembering what you are doing and searching for | Memory Work  Leisure activities | Body Function  Activity and Participation | b1 Mental Function  d8 Major life areas  d9 Community, social and civic life | b144 Memory Function  Work and employment (d840-d859)  d920 Recreation and leisure | d9209 Recreation and leisure, unspecified |
| *T02:"it's just, you're out. You're like, looking at your life passing by things that you were so easy before it becomes difficult and focus and things get challenging that were very easy"* | TBI | Change in identity post TBI; change in the difficulty level of activities impact on mobility | Self-identity Social life | Body Function  Activity and Participation | b1 Mental Functions  d9 Community, social and civic life | b180 Experience of self and time functions  d910 Community life | b1800 Experience of self  d9109 Community life, unspecified |
| *T02: "going specializing in any kind of sort of take socializing family gatherings with going to restaurants, cafes you know whether, it was in movies or anything that was loud, You know, concerts, small place music whenever all those things were very difficult for me"* | TBI | Sensitivity to stimulation, information, noise, light impact on socialization | Sensitivity to stimulation Social life | Body Function  Activity and Participation | b2 Sensory functions and pain  d9 Community, social and civic life | b240 Sensations associated with hearing and vestibular function d910 Community life | b2408 Sensations associated with hearing and vestibular function, other unspecified d9109 Community life, unspecified |
| *T02: "So all of my social life, family life, and my professional life are impacted"* | TBI | Social life, relationships impact post-TBI | Social life | Activity and Participation | d9 Community, social and civic life | d910 Community life | d9109 Community life, unspecified |
| *T03: “I can't read the kind of books I'm used to reading. It's actually very simple stuff. I can't remember anything"* | TBI | Reading comprehension, memory impacted post-TBI | Reading/Comprehension Leisure activities  Memory | Activity and Participation  Body Function | d1 Learning and applying knowledge d9 Community, social and civic life  b1 Mental Functions | Applying knowledge (d160-d179) d920 Recreation and leisure  b144 Memory Functions | d9209 Recreation and leisure, unspecified |
| *S03:“my life changed, I went from you know, jogging and yoga and being super active, to being even when I physically could do those things, petrified, petrified to do them”* | stroke | Psychological factors impact on mobility; change in identity | Psychological Sport | Activity and Participation  Body Function | d9 Community, social and civic life  b1 Mental Functions | d920 Recreation and leisure  b144 Memory functions | d9201 Sports |
| *T02: “I love my brain. I want it back, like my intellect; I think it was one of the best parts of me. I miss it"* | TBI | Change in cognitive abilities; changes and loss difficult post-TBI | Cognitive | Body Function | b1 Mental Functions | b164 Higher-level cognitive functions |  |
| *T03: "I want my brain back"* | TBI | Change in cognitive abilities; changes and loss difficult post-TBI | Cognitive | Body Function | b1 Mental Functions | b164 Higher-level cognitive functions  b144 Memory functions |  |
| *T02: "so I couldn't do the basic work of checking their work and sending emails that I've developed an anxiety and phobia around this and I had to give it up yesterday"* | TBI | Symptoms impacts on work and develop anxiety and fear | Anxiety Fear Work | Body Function  Activity and Participation | b1 Mental Functions  d8 Major life areas | b152 Emotional functions  Work and employment (d840-d859) | b1522 Range of emotion  b1528 Emotional functions, other specified (anxiety) |
| *T02: "I feel when I was after one month, I'm at home and then I feel me go into depression, I lost my job and there is no compensation after 7 months"* | TBI | Transition to home difficult; loss of job, finances also difficult; cascades to depression | Depression Work  Leisure activities Financial | Body Function  Activity and Participation  Environmental Factor | b1 Mental Functions  d8 Major life areas  d9 Community, social and civic life  e1 Products and Technology | b152 Emotional functions  Work and employment (d840-d859)  d920 Recreation and leisure  e165 Assets | b1528 Emotional functions, other specified (depression)  d9209 Recreation and leisure, unspecified  e 1650 Financial assets |
| *T02: "It's weird because I was self-isolating and then the pandemic happens like wow, like the world is itself isolating so yeah it's completely comprehensively, Impacted all of those aspects"* | TBI | Symptoms leads to self-isolation | Self-isolation | Personal Factor |  |  |  |
| *T03: "Stimulus any kind of stimulus visual, auditory everything gets to be too much very quickly so you know even visiting my home, my family"* | TBI | Sensitivity to stimulation, information, noise, light impact on mobility, relationships and family | Sensitivity to stimulation Visual Auditory Relationship with family | Body Function  Activity and Participation | b2 Sensory functions and pain  d7 Interpersonal interactions and relationships | b240 Sensations associated with hearing and vestibular function b210 Seeing functions b230 Hearing functions  d760 Family relationships | b2408 Sensations associated with hearing and vestibular function, other unspecified b2109 Seeing functions, unspecified b2309 Hearing functions, unspecified  d7609 Family relationships, other unspecified |
| *T03: "There aren't very many of us [i.e. family], but the TV and the conversations and the kids are making sounds and I just have to remove myself from the situation at some point and try to explain that I'm not being rude, I'm just overwhelmed with stimulus and I need less so"* | TBI | Sensitivity to stimulation, impacts relationship | Sensitivity to stimulation Family relationship | Body Function  Activity and Participation | b2 Sensory functions and pain  d7 Interpersonal interactions and relationships | b240 Sensations associated with hearing and vestibular function  d760 Family relationships | b2408 Sensations associated with hearing and vestibular function, other unspecified  d7609 Family relationships, other unspecified |
| *T03:"I was already self-isolating because I couldn't handle all the noise and the sounds and the vision and I also can't drive. I haven't been able to drive for my car for over a year now"* | TBI | Sensitivity to stimulation, information, noise, light & loss of driving impact on mobility | Sensitivity to stimulation Visual Auditory loss of driving licence | Body Function  Activity and Participation | b2 Sensory functions and pain  d4 Mobility | b240 Sensations associated with hearing and vestibular function b210 Seeing functions b230 Hearing functions  d475 Driving, | b2408 Sensations associated with hearing and vestibular function, other unspecified b2109 Seeing functions, unspecified b2309 Hearing functions, unspecified  d4759 Driving, unspecified |
| *T03: "And I found the winter, particularly difficult because you're I mean yourself isolating but you're really isolated in the wintertime, because nobody is outside. Even so, I don't know everybody's out there, living their lives and you just can't join in"* | TBI | Winter, snow, leads to self-isolation | Self-isolation Weather | Personal Factor  Environmental Factor | e2 Natural environment and human made changes to environment | e225 Climate | e 2255 Seasonal variation |
| **Theme 2. Assessment and intervention guidelines** | | | | | | | |
| **2.1. Finding common goals with patients** | | | | | | | |
| *C04:“will clients objectives first of all; if he is not going to use stairs because he is not going to use them, we are not going to use them”* | clinician | Mobility assessment methods: patients objectives/complain | Patient objective | Environmental Factor | e5 Services, systems and policies | e580 Health services, systems and policies | e5808 Health services, systems and policies, other specified (guidelines) |
| *C07:“also, patients’ complain is important, so if the number one complaint is dizziness, then we're going to focus on that. If its pain, we'll look at pain”* | clinician | Mobility assessment methods: patients objectives/complain | Patient objective | Environmental Factor | e5 Services, systems and policies | e580 Health services, systems and policies | e5808 Health services, systems and policies, other specified (guidelines) |
| *C02: “in OT, we would do cognitive screening [to assess cognitive impairment]”* | clinician | Mobility assessment methods; cognitive screening | Screening Cognitive | Environmental Factor  Body Function | e5 Services, systems and policies  b1 Mental Function | e580 Health services, systems and policies  b144 Memory functions | e5808 Health services, systems and policies, other specified (guidelines)  b1449 Memory functions, unspecified |
| *C07:“if he is able to carry things, does the laundry, able to prepare a meal, carry the pots. So, we can certainly do a little bit here [situational assessment]”* | clinician | Community-based ax: simulation / situational | Situational Assessment | Environmental Factor | e5 Services, systems and policies | e580 Health services, systems and policies | e5808 Health services, systems and policies, other specified (guidelines) |
| *C03:” part of the assessment is also establishing the persons self-reported difficulties, what they perceived to be difficult is a good starting to evaluate”* | clinician | Self-reported outcomes - as part of the assessment treatment cycle | Patient objective Self-reported | Environmental Factor | e5 Services, systems and policies | e580 Health services, systems and policies | e5808 Health services, systems and policies, other specified (guidelines) |
| *C07: “[we assess our patients focusing]More at the level of functional mobility, then more in the community and in using public transportation”* | clinician | Community-based assessment: simulation / situational | Situational assessment | Environmental Factor | e5 Services, systems and policies | e580 Health services, systems and policies | e5808 Health services, systems and policies, other specified (guidelines) |
| *C05: “In OT, in the back, [we go] shopping [with our patients] in the neighbourhood, we go with them to the bank, and for meal preparation at their home”* | clinician | Community-based assessment: simulation / situational | Situational assessment | Environmental Factor | e5 Services, systems and policies | e580 Health services, systems and policies | e5808 Health services, systems and policies, other specified (guidelines) |
| *C05: “Sometimes they don't achieve independency [while they are in rehab phase 2], so we train them in their neighbourhood or at least to make the trip from home to the outpatient clinic for their safety”* | clinician | Community-based assessment: simulation / situational | Situational assessment | Environmental Factor | e5 Services, systems and policies | e580 Health services, systems and policies | e5808 Health services, systems and policies, other specified (guidelines) |
| *C07: “we do a little preliminary assessment on the first day when the person is admitted; ....... after the first contact, I am going to go more towards the main problem [to evaluate]”* | clinician | Choose measures: depends on care pathway point; screening, patient's complain | Self-reported Screening | Environmental Factor | e5 Services, systems and policies | e580 Health services, systems and policies | e5808 Health services, systems and policies, other specified (guidelines) |
| *C03: “we have what the clients subjectively reports is their difficulty but we also have a professional responsibility to screen everything that they might not thought off”* | clinician | Assessment; difference btw what clients says and does or cannot do; words vs. behaviour; self-reported outcomes and screening | Self-reported Screening | Environmental Factor | e5 Services, systems and policies | e580 Health services, systems and policies | e5808 Health services, systems and policies, other specified (guidelines) |
| *C06: “sometimes the questionnaire gave us the wrong picture about the patient if we don’t ask him to do a task and observe”* | clinician | Caregiver/family: depends on deficits - may be better to ask caregivers/family, patient-reported outcomes, observation/ mixing between assessment methods | Self-reported Observation | Environmental Factor | e5 Services, systems and policies | e580 Health services, systems and policies | e5808 Health services, systems and policies, other specified (guidelines) |
| *C01:”there are objective assessment tools that we use in conjunction to observation to help guide me [to get the full picture while evaluating the patient in the clinic]"* | clinician | Observation/expert opinion; use of objective tools | Standardized measures Observation | Environmental Factor | e5 Services, systems and policies | e580 Health services, systems and policies | e5808 Health services, systems and policies, other specified (guidelines) |
| *C01: “we all use our clinical decision making, our experience to say what would be the most important tool to use”* | clinician | Integrate experience / clinical decision-making; lack of standard set of tools; clinical experience & judgment guiding what to assess and how to treat | Clinical judgement Expertise | Environmental Factor | e5 Services, systems and policies | e580 Health services, systems and policies | e5808 Health services, systems and policies, other specified (guidelines) |
| *C05: ‘if I have patient with mild TBI, It's more about vestibular problems, balance, and perceptual that can have an impact on how people move around in the community”* | clinician | Adapt assessment: depends on deficit - which guides assessment measures | Functional capacity | Environmental Factor | e5 Services, systems and policies | e580 Health services, systems and policies | e5808 Health services, systems and policies, other specified (guidelines) |
| *C02: “there is cognition and perception, also scanning and the vision aspects,[that need to be assessed]”* | clinician | Executive dis-function: poor perception, decision making, planning etc. impact on mobility, vision, safety, awareness | Screening Assessment Executive dysfunction  Safety  Vision | Body Function  Environmental Factors | b1 Mental function  b2 Sensory functions and pain  e5 Services, systems and policies | b164 Higher-level cognitive functions b210 Seeing functions  e530 Utilities services, systems and policies  e580 Health services, systems and policies | b2101 Visual field functions  e5308 Utilities services, systems and policies, other specified (Safety standards for individuals with ABI)  e5808 Health services, systems and policies, other specified (guidelines) |
| *C03:“we need to look at flexibility, we need to look at pain, spasticity, In terms of things that we are going to change sure, coordination, we also, want to look at their endurance, strength, balance”* | clinician | Assessment; pain, spasticity, coordination, endurance, strength, balance | Standardized measures Observation  Pain Spasticity Coordination Endurance Strength Balance | Environmental Factor  Body Function | e5 Services, systems and policies  b2 Sensory Functions and Pain  b4 Functions of the cardiovascular, haematological, immunological and respiratory systems  b7 Neuromusculoskeletal and movement-related functions | e580 Health services, systems and policies Pain (b280-b289)  b455 Exercise tolerance functions  b735 Muscle tone functions  b235 Vestibular functions | e5808 Health services, systems and policies, other specified (guidelines)  b4550 General physical endurance  b7358 Muscle tone functions, other specified (Spasticity)  b2358 Vestibular functions, other specified (Balance, coordination) |
| *C07: “we need to see how the person transfers; does he or she need to have a wheelchair? Does he or she need an aid? Walker, orthotic, a splint, depending on the condition”* | clinician | Choose measures: depends on condition/deficit; evaluate mobility: observation | Observation | Environmental Factor | e5 Services, systems and policies | e580 Health services, systems and policies | e5808 Health services, systems and policies, other specified (guidelines) |
| *C03:“we need to evaluate different elements to have a set to get a sense what is the impairments and handicaps situation and everything the person is doing but also we are able to determine a month or 2 months later has there been a change”* | clinician | Mobility assessment methods; time series, baseline, post to track the changes | Standardized measures /responsiveness | Environmental Factor | e5 Services, systems and policies | e580 Health services, systems and policies | e5808 Health services, systems and policies, other specified (guidelines) |
| *C04: “clinical judgment and the degree of the sensitivity to change to target functional abilities in the community”* | clinician | Priorities; EBP - clinical judgment, responsiveness, community-based | Clinical judgment Standardized measures | Environmental Factor | e5 Services, systems and policies | e580 Health services, systems and policies | e5808 Health services, systems and policies, other specified (guidelines) |
| *C05:“objective evaluation it can also help us to highlight other deficits that the person has”* | clinician | Mobility assessment: objective | Standardized measures | Environmental Factor | e5 Services, systems and policies | e580 Health services, systems and policies | e5808 Health services, systems and policies, other specified (guidelines) |
| *C04: “use the scores and also the clinical judgment, I think it is a combination of both”* | clinician | Score - as part of evidence based practice | Clinical judgment Standardized measures | Environmental Factor | e5 Services, systems and policies | e580 Health services, systems and policies | e5808 Health services, systems and policies, other specified (guidelines) |
| *C03: “it [objective tool] helps to be objective because we get invested in the person and we want them to get better, sometimes we have to take a step back and say no they did not change”* | clinician | Score - as a means to be objective/professional | Clinical judgment Standardized measures | Environmental Factor | e5 Services, systems and policies | e580 Health services, systems and policies | e5808 Health services, systems and policies, other specified (guidelines) |
| *C02: “I would say that the only time I go with score it is for driving because I cannot go and evaluate a driving by a mise en situation [i.e. simulation]”* | clinician | Score - limits to using score when unable to assess directly | Standardized measures Driving | Environmental Factor  Activity and Participation | e5 Services, systems and policies  d4 Mobility | e580 Health services, systems and policies  d475 Driving | e5808 Health services, systems and policies, other specified (guidelines)  d4759 Driving, unspecified |
| *C01: “the uses of the scores to help me when the client who doesn’t see their deficits, so I use the objective tool as an argument to support my recommendation”* | clinician | Score - as an educational tool for patient, support clinicians recommendations | Standardized measures | Environmental Factor | e5 Services, systems and policies | e580 Health services, systems and policies | e5808 Health services, systems and policies, other specified (guidelines) |
| *C03: “these numbers are more not what to treat but it is that they don’t want treatment”* | clinician | Score-tracking change, decision making | Clinical judgment Standardized measures | Environmental Factor | e5 Services, systems and policies | e580 Health services, systems and policies | e5808 Health services, systems and policies, other specified (guidelines) |
| *C05: “for us the FIM is really important to do because it's made the recommendation”* | clinician | Choose measures: best practices (decision making) | Clinical judgment Standardized measures | Environmental Factor | e5 Services, systems and policies | e580 Health services, systems and policies | e5808 Health services, systems and policies, other specified (guidelines) |
| *C07: “That's it, often the coordinator will really do an initial meeting that gives us a fairly global picture from the start”* | clinician | Initial assessment, comprehensive ax at referral | Self-reported | Environmental Factor | e5 Services, systems and policies | e580 Health services, systems and policies | e5808 Health services, systems and policies, other specified (guidelines) |
| *C05: Well, I don't know, PRO might highlight some areas that I'm going to want to go deeper into”* | clinician | Types of assessment; patient-reported vs. clinicians-reported | Self-reported | Environmental Factor | e5 Services, systems and policies | e580 Health services, systems and policies | e5808 Health services, systems and policies, other specified (guidelines) |
| *C06: "we based our interventions based on patients' objectives and our clinical judgments"* | clinician | Blending client with goals with clinical assessment / judgement and expertise | Patient objective Clinical judgment | Environmental Factor | e5 Services, systems and policies | e580 Health services, systems and policies | e5808 Health services, systems and policies, other specified (guidelines) |
| *C05: “It's rare, it's going to be more in terms of their perception of their energy level, their physical endurance, but otherwise it's pretty rare for me to use self-reported questionnaires”* | clinician | Types of assessment; patient-reported vs. clinicians-reported; balance between patient-reported and clinical observation and assessment | Self-reported/limited | Environmental Factor | e5 Services, systems and policies | e580 Health services, systems and policies | e5808 Health services, systems and policies, other specified (guidelines) |
| *C07: “using a questionnaire, it's still too much at the beginning if there is a bit of aphasia in there, comprehension problem, are not able to read, or you know they are able to just say simple answers”* | clinician | Timing of when to use Ax tools; client deficits impact on usefulness/feasibility of assessment | Self-reported  aphasia | Environmental Factor  Body Function | e5 Services, systems and policies  b1 Mental Functions | e580 Health services, systems and policies  b167 Mental functions of language | e5808 Health services, systems and policies, other specified (guidelines)  b1688 Mental functions of language, other specified (aphasia) |
| *C05: “for example, people with hemianopsia, often they really need to be supervised to fill out the questionnaire”* | clinician | Visual deficits may impact on functioning; need help with assessment | Self-reported  Cognition | Environmental Factor  Body Function | e5 Services, systems and policies  b1 Mental Function | e580 Health services, systems and policies  b144 Memory functions | e5808 Health services, systems and policies, other specified (guidelines)  b1449 Memory functions, unspecified |
| *C05: “Filling out a questionnaire alone is often difficult”* | clinician | Limited functioning may impact on completing assessment; lack of support network | Self-reported/limited  Cognition | Environmental Factor  Body Function | e5 Services, systems and policies  b1 Mental Function | e580 Health services, systems and policies  b144 Memory functions | e5808 Health services, systems and policies, other specified (guidelines) b1449 Memory functions, unspecified |
| *C06: “the problem is that some patients don’t see their deficits, so when we ask them what are the things that you cannot do? The answer is I can do everything, everything is fine”* | clinician | Lack of awareness may impede completion of assessment | Self-reported/limited | Environmental Factor | e5 Services, systems and policies | e580 Health services, systems and policies | e5808 Health services, systems and policies, other specified (guidelines) |
| *C06: “if we ask them about their goals, they answer to get back home as soon as possible, it is only because they don't see their deficit”* | clinician | Lack of awareness may impede completion of assessment | Self-reported/limited | Environmental Factor | e5 Services, systems and policies | e580 Health services, systems and policies | e5808 Health services, systems and policies, other specified (guidelines) |
| *C04: “a member of family or a caregiver can fill the questionnaire for them”* | clinician | Using proxies to help with assessment | Proxy assessment Cognition | Environmental Factor  Body Function  Activity and Participation | e5 Services, systems and policies  b1 Mental Function  b7 Interpersonal interactions and relationships | e580 Health services, systems and policies b144 Memory functions  d760 Family relationships | e5808 Health services, systems and policies, other specified (guidelines) b1449 Memory functions, unspecified  d7608 Family relationships, other specified (family support) |
| *C03: “what are the red flags that require an intervention? Fear, problems with vision, pain, depression, fatigue, dizziness, headaches, if they mention any of these problems it may require other evaluations”* | clinician | Priorities; client-centred; assessment priorities; indicators that require interventions | Red flag indicators | Environmental Factor | e5 Services, systems and policies | e580 Health services, systems and policies | e5808 Health services, systems and policies, other specified (guidelines) |
| *C05: “take into account the red flags especially for older adults who have high risk of falling”* | clinician | Risks during community-based rehab impact on mobility | Red flag indicators | Environmental Factor | e5 Services, systems and policies | e580 Health services, systems and policies | e5808 Health services, systems and policies, other specified (guidelines) |
| *C05: “I think yes the objective tests are very valid but how the person is functioning is much important to decide the intervention”* | clinician | Combining objective ax tools with observation, situational, simulation, patient-reported outcomes | Standardized measures  Situational assessment | Environmental Factor | e5 Services, systems and policies | e580 Health services, systems and policies | e5808 Health services, systems and policies, other specified (guidelines) |
| *C01: “We use a questionnaire to predict how the patient can get back to work considering the number of symptoms that they have when they began”* | clinician | Ax to predict recovery; planning for return to work, mobility | Self-reported | Environmental Factor | e5 Services, systems and policies | e580 Health services, systems and policies | e5808 Health services, systems and policies, other specified (guidelines) |
| *C01: “When the patient begins with us, he fill a number of questionnaire. One will be about self-efficacy, and then we re-do the questionnaire at the end of the process”* | clinician | Stages of assessment; pre-post; types of ax tools | Self-reported/first contact | Environmental Factor | e5 Services, systems and policies | e580 Health services, systems and policies | e5808 Health services, systems and policies, other specified (guidelines) |
| *C02: “if the inpatient and the acute outpatient and the chronic outpatient all use the same test, then we can track measures across the time, but everybody uses different things, so it is hard to see if there is been progress”* | clinician | System wide policy; use common assessment tools to enable comparison, tracking progress | Standardized measures /consistency | Environmental Factor | e5 Services, systems and policies | e580 Health services, systems and policies | e5808 Health services, systems and policies, other specified (guidelines) |
| *C06: “I find using the scores much more as a way to see the progression than to use it as a way to establish the plan as you know”* | clinician | Scores used to see responsiveness; scores to see change/progress; | Standardized measures /responsiveness | Environmental Factor | e5 Services, systems and policies | e580 Health services, systems and policies | e5808 Health services, systems and policies, other specified (guidelines) |
| *C05: “the objective tests help me to have a global picture at the beginning and make recommendation to discharge the patient”* | clinician | Objective assessment tools facilitate clear understanding of rehab treatment plan & discharge for client | Standardized measures /global and recommendation | Environmental Factor | e5 Services, systems and policies | e580 Health services, systems and policies | e5808 Health services, systems and policies, other specified (guidelines) |
| *C07: I think in rehab it's a lot of sharing between all the stakeholders in relation to each other”* | clinician | Interdisciplinary approach to rehab; impact on mobility | Interdisciplinary shared decision making | Activity and Participation | d3 Communication | Conversation and use of communication devices and techniques (d350- d369) | Activity and Participation |
| *C07: “I think the richness of teamwork is to exchange a lot of observations about the same patient”* | clinician | Interdisciplinary approach to rehab; share info/data; impact on mobility | Interdisciplinary shared decision making | Activity and Participation | d3 Communication | Conversation and use of communication devices and techniques (d350- d369) | Activity and Participation |
| *C06: “Yes, the interdisciplinary work is helping us a lot”* | clinician | Interdisciplinary approach to rehab; impact on mobility | Interdisciplinary shared decision making | Activity and Participation | d3 Communication | Conversation and use of communication devices and techniques (d350- d369) | Activity and Participation |
| *C07: “it's sure that we will have the objectives of the intervention plan. Also, it's true that we have our own objectives, you know, disciplinary objectives”* | clinician | Discipline specific treatment plan integrated with interdisciplinary treatment plan | Interdisciplinary shared decision making | Activity and Participation | d3 Communication | Conversation and use of communication devices and techniques (d350- d369) | Activity and Participation |
| *C07: “the intervention plan depends if it's the disciplinary or interdisciplinary plan”* | clinician | Treatment plan depends on resources, team, context; disciplinary vs. interdisciplinary | Interdisciplinary shared decision making | Activity and Participation | d3 Communication | Conversation and use of communication devices and techniques (d350- d369) | Activity and Participation |
| *C05: “to see the contribution of the physio assessment plus the neuropsychological assessment plus the ergo assessment at the same level that's what will have the greatest impact on our intervention plan”* | clinician | Discipline specific treatment plan integrated with interdisciplinary treatment plan | Interdisciplinary shared decision making | Activity and Participation | d3 Communication | Conversation and use of communication devices and techniques (d350- d369) | Activity and Participation |
| *C05: “to have goals that are achievable across the team to meet patient’s needs”* | clinician | Client-centred, goal-setting; Discipline specific treatment plan integrated with interdisciplinary treatment plan | Interdisciplinary shared decision making | Activity and Participation | d3 Communication | Conversation and use of communication devices and techniques (d350- d369) | Activity and Participation |
| *C07: “It's good to be more specific in each discipline, it's important to be more specific in each discipline too”* | clinician | Treatment plan depends on resources, team, context; disciplinary vs. interdisciplinary | Interdisciplinary shared decision making | Activity and Participation | d3 Communication | Conversation and use of communication devices and techniques (d350- d369) | Activity and Participation |
| *C07: “There's a lot of exchange between the stakeholders”* | clinician | Communication between stakeholders | Interdisciplinary shared decision making | Activity and Participation | d3 Communication | Conversation and use of communication devices and techniques (d350- d369) | Activity and Participation |
| *C07: “When you see a decrease in physical capacity and then after that in ergo they see the impact of a lifestyle habit, lack of endurance or, lack of strength in one leg while going down the stairs of the supermarket that doesn't have ramp, you know, we're going to exchange the information a lot”* | clinician | Sharing info: key changes in behaviour and function communicated with team | Interdisciplinary shared decision making | Activity and Participation | d3 Communication | Conversation and use of communication devices and techniques (d350- d369) | Activity and Participation |
| *T04: "What are those steps and How to apply them, you really need a specialist to really get in there and focus and help you Improve on very specific things and how to do those things"* | TBI | Tailored rehab to specific deficits; need specialists to help recover | Uncertainty | Not defined |  |  |  |
| *C03: “we have to understand the client’s reality before we start doing basically anything and taking the time to ask them even before evaluating which is a part of the assessment? What are their difficulties? And what are their priorities”* | clinician | Priorities; client-centred | Patients objectives | Environmental Factor | e5 Services, systems and policies | e580 Health services, systems and policies | e5808 Health services, systems and policies, other specified (guidelines) |
| *C07: “the intervention is more functional based on the patient’s goals”* | clinician | Treatment matched to client goals; client-centred | Patients objectives | Environmental Factor | e5 Services, systems and policies | e580 Health services, systems and policies | e5808 Health services, systems and policies, other specified (guidelines) |
| *C07: “for example, the person's objective will be to participate in a community café in their neighborhood, so it is necessary to work on his endurance and balance to go there”* | clinician | Client-centred; client goals and tailoring intervention and treatment plan to client goals | Patients objectives | Environmental Factor | e5 Services, systems and policies | e580 Health services, systems and policies | e5808 Health services, systems and policies, other specified (guidelines) |
| *C07: “the goal at the end of the line is that he is able to meet his essential needs”* | clinician | Client-centred; start with basic needs and move up to work, leisure, relationships in treatment plan | Patients objectives | Environmental Factor | e5 Services, systems and policies | e580 Health services, systems and policies | e5808 Health services, systems and policies, other specified (guidelines) |
| **2.2. Challenges clinicians faced when they evaluate mobility** | | | | | | | |
| *C04: “Berg is really good but it took 30 minutes, the BESTest took 45 minutes to finish, I mean there is too many things to look at instead of using a tool”* | clinician | Assessment; complicated - no right answer; Limited time and resources | Standardized measures / limited | Environmental Factor | e5 Services, systems and policies | e580 Health services, systems and policies | e5808 Health services, systems and policies, other specified (guidelines) |
| *C06: “yes, because some of these assessments are not transferable to real life”* | clinician | Standard assessment along with situational / simulation assessment; observation ax to assess mobility | Standardized measures /limited | Environmental Factor | e5 Services, systems and policies | e580 Health services, systems and policies | e5808 Health services, systems and policies, other specified (guidelines) |
| *C05: “sometimes in the community, its hard to use a standardize measure to evaluate mobility because of different environment, so it is more functional”* | clinician | Which mobility measure/tool to use varies with context/environment | Standardized measures /limited | Environmental Factor | e5 Services, systems and policies | e580 Health services, systems and policies | e5808 Health services, systems and policies, other specified (guidelines) |
| *C03:“fatigue is another obstacle if you have to do the BORG over three visits”* | clinician | Assessment: fatigue; limited resources; burden on patient | Standardized measures / limited | Environmental Factor | e5 Services, systems and policies | e580 Health services, systems and policies | e5808 Health services, systems and policies, other specified (guidelines) |
| *C03:“we have got an hour and we have to choose one or two tests, hopefully do some treatments and teach them something”* | clinician | Assessment treatment cycle limits of time | Standardized measures / limited | Environmental Factor | e5 Services, systems and policies | e580 Health services, systems and policies | e5808 Health services, systems and policies, other specified (guidelines) |
| *C03: “there is a limit of how much because there is no enough time to assess [if] they can swallow, they can eat, they can dress, and they can walk themselves”* | clinician | Limits to discharge planning; assess safety, mobility, activities of daily living | Standardized measures / limited | Environmental Factor | e5 Services, systems and policies | e580 Health services, systems and policies | e5808 Health services, systems and policies, other specified (guidelines) |
| *C03:“what we need to know and you know in terms of research questions ,what are the top 5, top 10 tests that are going to be helpful”* | clinician | Assessment methods; limited time and resources; limited choices in what tools to use | Standardized measures / top | Environmental Factor | e5 Services, systems and policies | e580 Health services, systems and policies | e5808 Health services, systems and policies, other specified (guidelines) |
| *C03: “I think we have to get down to a short list of at least covering the different domains in physiotherapy”* | clinician | Limited time; lack of guidelines, limited choice in what tool to use | Standardized measures / top | Environmental Factor | e5 Services, systems and policies | e580 Health services, systems and policies | e5808 Health services, systems and policies, other specified (guidelines) |
| *C06: “there's no systematic way to choose the measures”* | clinician | Choose measures: no systematic method | Standardized measures / limited | Environmental Factor | e5 Services, systems and policies | e580 Health services, systems and policies | e5808 Health services, systems and policies, other specified (guidelines) |
| *C01:“another barrier for sure is the client themselves in term of fear, do they trust you, or even if they trust you are they able to put themselves in a situation where they are challenged”* | clinician | Assessment; self-awareness, avoidance/fear, trust in clinician/barriers to evaluate mobility | Trust | Body Function | b1 Mental Functions | b153 Emotional Functions | b1522 Range of emotion |
| *C01:“[Clients were] basically home bound in winter because either they don’t have the confidence or just very difficult to get out in wheelchair, probably a combination of the two?”* | clinician | Fear and avoidance, no confidence in ability/access in winter (using the wheelchair) | Fear Confidante Weather | Environmental Factors  Body Function | e2 Natural environment and human made changes to environment  b1 Mental Functions | e225 Climate  b153 Emotional Functions  b126 Temperament and personality functions | e 2255 Seasonal variation  b1522 Range of emotion  b1266 Confidence |
| *C07:“other people that it's really a lack of endurance, and then the use of walking aid in winter weather may results into falling”* | clinician | determining factor is physical; cognitive may be more serious; clinical judgment | Fatigue Weather | Body Function  Environmental Factors | b4 Functions of the cardiovascular, haematological, immunological and respiratory systems  e2 Natural environment and humanmade changes to environment | b455 Exercise tolerance functions  e225 Climate | b4552 Fatigability  e 2255 Seasonal variation |
| *C05: “Another aspect which is a barrier to mobility is the use of alcohol or drugs”* | clinician | Mobility assessment challenges: alcohol or drug use | Alcohol/Drug | Body Function | b1 Mental Functions | b 110 Consciousness functions | b1102 Quality of consciousness |
| *C05:“We have clients with a problem of abusive consumption, when they return home and resume their consumption, they will have falls”* | clinician | Mobility assessment challenges: alcohol or drug use | Alcohol/Drug | Body Function | b1 Mental Functions | b 110 Consciousness functions | b1102 Quality of consciousness |
| *C07:“when we talk about cognitive versus physical, it depends on the clients, there are clients for whom the cognitive dominates, which make them unsafe to cross the street, they don't orient themselves in their neighbourhood”* | clinician | Determining factor is physical; cognitive may be more serious; clinical judgment | Cognitive  Safety | Environmental Factor  Body Function | e5 Services, systems and policies  b1 Mental Function | e530 Utilities services, systems and policies   b144 Memory functions  b164 Higher-level cognitive functions | e5308 Utilities services, systems and policies, other specified (Safety standards for individuals with ABI) |
| *C03:“the lack of fear and the lack of awareness often the patients with cognitive problems will they are not limited by fear”* | clinician | Executive dysfunction: poor perception, decision making, planning etc. impact on mobility, safety perception, awareness, lack of fear | Cognitive  Safety | Environmental Factor  Body Function | e5 Services, systems and policies  b1 Mental Function | e530 Utilities services, systems and policies   b144 Memory functions  b164 Higher-level cognitive functions | e5308 Utilities services, systems and policies, other specified (Safety standards for individuals with ABI) |
| *C03:“they over estimate their abilities”* | clinician | Executive dysfunction: poor perception, decision making, planning etc. impact on mobility, safety perception, awareness, lack of fear | Cognitive  Safety | Environmental Factor  Body Function | e5 Services, systems and policies  b1 Mental Function | e530 Utilities services, systems and policies   b144 Memory functions  b164 Higher-level cognitive functions | e5308 Utilities services, systems and policies, other specified (Safety standards for individuals with ABI) |
| *C08:“feeling safe in the environment”* | clinician | Mobility assessment challenge in the community: safety issues | Safety | Environmental Factor | e5 Services, systems and policies | e530 Utilities services, systems and policies | e5308 Utilities services, systems and policies, other specified (Safety standards for individuals with ABI) |
| *C03:“safety, awareness [and] being able to cross the street”* | clinician | Executive dysfunction: poor perception, decision making, planning, self-awareness etc. impact on mobility, safety | Cognitive  Safety | Environmental Factor  Body Function | e5 Services, systems and policies  b1 Mental Function | e530 Utilities services, systems and policies   b144 Memory functions  b164 Higher-level cognitive functions | e5308 Utilities services, systems and policies, other specified (Safety standards for individuals with ABI) |
| *C03:“there is basic safety things that sometimes will people be more impulsive in lack of judgment and be attentive”* | clinician | Executive dysfunction: poor perception, decision making, planning, self-awareness etc. impact on mobility, safety | Cognitive  Safety | Environmental Factor  Body Function | e5 Services, systems and policies  b1 Mental Function | e530 Utilities services, systems and policies   b144 Memory functions  b164 Higher-level cognitive functions | e5308 Utilities services, systems and policies, other specified (Safety standards for individuals with ABI) |
| *C13:“there is an issue of balance and coordination, and many of these patients have complex problems because it might go with vision problem”* | clinician | Mobility assessment challenge in the community: balance, coordination, vision issues | Balance Vision | Body Function | b2 Sensory functions and pain | b235 Vestibular Functions  b210 Seeing Functions | b2358 Vestibular functions, other specified (Balance, coordination)  b2101 Visual field functions |
| *C14: “They [TBI] have difficulty to integrate what they feel and what they see”* | clinician | Mobility assessment challenge in the community: integration between themselves and the environment | Cognitive  Safety | Environmental Factor   Body Function | e5 Services, systems and policies   b1 Mental Function | e530 Utilities services, systems and policies  b144 Memory functions  b164 Higher-level cognitive functions | e5308 Utilities services, systems and policies, other specified (Safety standards for individuals with ABI) |
| *C14:“It is more psychologist, so the person needs to regain confidence”* | clinician | Mobility assessment challenge in the community: regain confidence | Cognitive  Safety Confidence | Environmental Factor  Body Function | e5 Services, systems and policies  b1 Mental Function | e530 Utilities services, systems and policies   b144 Memory functions  b164 Higher-level cognitive functions  b126 Temperament and personality functions | e5308 Utilities services, systems and policies, other specified (Safety standards for individuals with ABI)  b1266 Confidence |
| *C13:“basically, there is a link between confidence and anxiety”* | clinician | Mobility assessment challenge in the community: regain confidence | Confidence Anxiety | Body Function | b1 Mental Functions | b126 Temperament and personality functions  b152 Emotional functions | b1266 Confidence  b1522 Range of emotion |
| **2.3. Engaging the patient and considering their perspectives in their care** | | | | | | | |
| *S02:“I was asking why you are doing this test or whatever this function and what”* | stroke | Patients are asking questions related to the purpose of the evaluation/patient centred care | Patient engagement | Environmental Factor | e5 Services, systems and policies | e580 Health services, systems and policies | e5808 Health services, systems and policies, other specified (guidelines) |
| *S02: “I would actually stop at the beginning and ask what do you want to gain out of this, like what's the purpose of it”* | stroke | Patients are asking questions related to the purpose of the evaluation/patient centred care | Patient engagement | Environmental Factor | e5 Services, systems and policies | e580 Health services, systems and policies | e5808 Health services, systems and policies, other specified (guidelines) |
| *S02 “they have a protocol, and so basically I was applying that to everything that had to be done was like OK, stop what are we doing here?”* | stroke | Patients are asking questions related to the purpose of the evaluation/strict protocol followed while ax and treatment/patient centred care | Patient engagement | Environmental Factor | e5 Services, systems and policies | e580 Health services, systems and policies | e5808 Health services, systems and policies, other specified (guidelines) |
| *S05: “I was a little more cautious and not just doing likely whatever they said like”* | stroke | Patients are asking questions related to the purpose of the evaluation/strict protocol followed while ax and treatment/patient centred care | Patient engagement | Environmental Factor | e5 Services, systems and policies | e580 Health services, systems and policies | e5808 Health services, systems and policies, other specified (guidelines) |
| *S03:“I assumed that's what should happen because, you know this is how they going to treat me”* | stroke | No questions is asked assuming that the healthcare provider knows exactly what to do/patient centred care | Patient engagement | Environmental Factor | e5 Services, systems and policies | e580 Health services, systems and policies | e5808 Health services, systems and policies, other specified (guidelines) |
| *S03 “I take it to mean you know what's best for me because you've seen this before, and you know how to handle it”* | stroke | No questions is asked assuming that the healthcare provider knows exactly what to do/patient centred care | Patient engagement | Environmental Factor | e5 Services, systems and policies | e580 Health services, systems and policies | e5808 Health services, systems and policies, other specified (guidelines) |
| *S05:“I learned there to say no to certain things coz they would really bad decision”* | stroke | Patients learn how to say "no" for certain ax and treatment/bad decisions are taken by the healthcare provider/patient-centred care/Allen Edwards hospital | Patient engagement | Environmental Factor | e5 Services, systems and policies | e580 Health services, systems and policies | e5808 Health services, systems and policies, other specified (guidelines) |
| **Theme 3. Support Network** | | | | | | | |
| **3.1. Caregiver support** | | | | | | | |
| *C06:“a family member or a caregiver can help especially for patients with cognitive issues”* | clinician | Caregiver/family: depends on deficits/cognition - may be better to ask caregivers/family | Cognition Family/Caregiver | Body Function  Environmental Factors | b1 Mental Function  e3 Support and relationships | b144 Memory functions  e310 Immediate family | b1449 Memory functions, unspecified |
| *C06:“by some discussion with the patient's family, we can point into their deficits”* | clinician | Caregiver/family: depends on deficits/cognition - may be better to ask caregivers/family | Cognition Family/Caregiver | Body Function  Environmental Factors | b1 Mental Function  e3 Support and relationships | b144 Memory functions  e310 Immediate family | b1449 Memory functions, unspecified |
| *C01:“if the clients has cognitive issues he might want to get the family members involved as well to validate what he is saying or maybe give extra information”* | clinician | Caregiver/family: depends on deficits/cognition - may be better to ask caregivers/family | Cognition Family/Caregiver | Body Function  Environmental Factors | b1 Mental Function  e3 Support and relationships | b144 Memory functions  e310 Immediate family | b1449 Memory functions, unspecified |
| *C04:“aphasia is a big topic, can we be able to talk to the person”* | clinician | Caregiver/family: depends on deficits/aphasia - may be better to ask caregivers/family | Aphasia Family/Caregiver | Body Function  Environmental Factors | b1 Mental Function  e3 Support and relationships | b167 Mental functions of language  e310 Immediate family | b1688 Mental functions of language, other specified (Aphasia) |
| *C01:“a lot of people maybe it is a new thing that their loved one is using a wheelchair”* | clinician | Adjustment to new life | Family/Caregiver | Environmental Factors | e3 Support and relationships | e310 Immediate family | Environmental Factors |
| *T04:“my husband is give me too much help for improve my mental stress"* | TBI | Family / spousal support helps with stress; difficult to navigate system - paperwork and files spread across many points | Family/Caregiver | Environmental Factors | e3 Support and relationships | e310 Immediate family | Environmental Factors |
| *T04:"I think the psychologist and the support from your family is more effective"* | TBI | Family support | Family/Caregiver | Environmental Factors | e3 Support and relationships | e310 Immediate family | Environmental Factors |
| *T04:"my husband gave me a lot of time and discussion and relaxations"* | TBI | Family/spousal support helps | Family support | Environmental Factors | e3 Support and relationships | e310 Immediate family | Environmental Factors |
| *T04: “a family support is very, very healthy for me because I have two sons and my husband in my home, my son is 14 year old and then my son and my husband all the time is with me [to support me]”* | TBI | Family / spousal support helps | Family support | Environmental Factors | e3 Support and relationships | e310 Immediate family | Environmental Factors |
| *T04:"all the time my husband and my son give me a boost like going outside and met some family members over the phone"* | TBI | Family support, spousal support help with mental health | Family support Mental health | Environmental Factors  Body Function | e3 Support and relationships  b1 Mental Functions | e310 Immediate family  b199 Mental Functions, unspecified |  |
| *S02: “Luckily she had the day off [his caregiver], so she can help me out for certain things or would I can't get something”* | stroke | Caregiver support useful and required for mobility and community participation | Family support | Environmental Factors | e3 Support and relationships | e310 Immediate family |  |
| *S03:“You know if you're lucky enough to have that for your own cognitive ability to advocate for yourself, that's one and then if you have people that you're surrounded by who can advocate for you as well, not everybody's that fortunate also”* | stroke | Cognitive abilities and family support | Family support Cognition | Environmental Factors  Body Function | e3 Support and relationships  b1 Mental Functions | e310 Immediate family  b144 Memory functions |  |
| *S02: “No that's for sure I go to the hospital I dropped my arms [to his wife] and just let her go, because she knows the system”* | stroke | Caregiver/family support | Family support | Environmental Factors | e3 Support and relationships | e310 Immediate family |  |
| **3.2. Providers support** | | | | | | | |
| *T02:"I thought the team of the xxx was very good. They were on board, I felt finally really supported"* | TBI | Institutional support; great services | Provider support | Environmental Factors | e3 Support and relationships | e325 Acquaintances, peers, colleagues, neighbours and community members |  |
| *T02:"I found everyone to be very kind and understanding because I've never had a concussion and I it's very confusing"* | TBI | Institutional support; great services | Provider support | Environmental Factors | e3 Support and relationships | e325 Acquaintances, peers, colleagues, neighbours and community members |  |
| *T01:"So it's just having people believe you and support you and understand the difficulties that you're going through finding those health professionals, especially at the neuroscientist kind of level”* | TBI | Support is important, listening, understanding is important | Provider support | Environmental Factors | e3 Support and relationships | e325 Acquaintances, peers, colleagues, neighbours and community members |  |
| *C13: “we work with the families, so it is important to get their point of view and their input and to help them to cope into the situation to help the patient”* | clinician | Working with family/caregivers to facilitate coping styles | Family/Caregiver Coping style | Environmental Factors | e3 Support and relationships | e310 Immediate family |  |
| **3.3. Community Support** | | | | | | | |
| *S06:“Like I was just saying, people at the hospital, homes, they think you are stupid, but the public in general, they pretty nice about it, they come and ask, can we help you?”* | stroke | Perceptions of people at institutions vs. community and stigma associated with stroke; impacts mobility | Community support Stigma | Environmental Factors | e3 Support and relationships | e325 Acquaintances, peers, colleagues, neighbours and community members |  |
| *S06:“I find one thing quite annoying is that when you are put around in a wheelchair, people look at you, and some taxi drivers, they think your brain is gone, they think you stupid”* | stroke | Stigma associated with stroke; visible disability and using a wheelchair; possible avoidance of use of services | Community support  Stigma | Environmental Factors | e3 Support and relationships | e325 Acquaintances, peers, colleagues, neighbours and community members |  |
| *S04: “when I first get my cane, after I get out at Jean Coutu with my dad, purchase a cane. and standing in line trying to get out, the younger people, they get out of your way, they help you a lot”* | stroke | Visible disability via a cane can be perceived positively by younger members community; they help with mobility | Community support | Environmental Factors | e3 Support and relationships | e325 Acquaintances, peers, colleagues, neighbours and community members |  |
| *S04: “the elder people didn't, they push you like they couldn't give a hood”* | stroke | Visible disability via a cane can be perceived positively by younger members community; they help with mobility | Community support | Environmental Factors | e3 Support and relationships | e325 Acquaintances, peers, colleagues, neighbours and community members |  |
| *C05:“knowing what the best ways to accompany them to be mobile in community as possible when they can't resume driving for example”* | clinician | Community supports to facilitate mobility | Community support | Environmental Factor | e5 Services, systems and policies | e575 General social support services, systems and policies | e 5758 General social support services, systems and policies, other specified (community) |
| *C05: “[the support services are missing, especially when the patient] is not [obtaining] the necessary balance, the necessary endurance or because it is not well oriented and safe to cross the street”* | clinician | Community-based - lack of support services; difficult to plan | Community support | Environmental Factor | e5 Services, systems and policies | e575 General social support services, systems and policies | e 5758 General social support services, systems and policies, other specified (community) |
| *C06: “people who don't have caregivers around them who can't take the car, who are not independent to take public transit [also, have a problem to get access to the outpatient services]”* | clinician | community-based - lack of support services; difficult to plan | Community support | Environmental Factor | e5 Services, systems and policies | e575 General social support services, systems and policies | e 5758 General social support services, systems and policies, other specified (community) |
| *C06: “in stroke, there's no services, you know, they end up after that feeling like there's no one left, there is no services that can be provided for them”* | clinician | Community-based - lack of services; difficult to plan | Community support | Environmental Factor | e5 Services, systems and policies | e575 General social support services, systems and policies | e 5758 General social support services, systems and policies, other specified (community) |
| **Theme 4. Uncertainty about symptoms and recovery** | | | | | | | |
| *T02: “Will I ever get back to ‘normal’?, Are the changes to my cognitive functioning permanent?”* | TBI | Uncertainty of deficits and recovery limits mobility | Uncertainty  Recovery Symptom | Not covered |  |  |  |
| *T01:“It's like you just never know how long you're going to be well last, you know, It was tough"* | TBI | Uncertainty about severity of deficits impact on participation | Uncertainty  Symptom  Recovery Psychological | Not covered  Body Function | b1 Mental Functions | b199 Mental Functions, unspecified |  |
| *T01:"feel like I can kind of live with symptoms, little bit, but it's like, am I always going to have them"* | TBI | Uncertainty of progress and recovery make it difficult; | Uncertainty  Symptom  Recovery | Not covered |  |  |  |
| *T01: "I'm mostly resolved from it, but there's still some little symptoms and you kind of wonder, is that going to last forever or if there are ways to kind of do right now, especially because of all COVID as we don’t have any therapies really like accessible "* | TBI | Uncertainty of progress and recovery make it difficult; lack of services due to COVID-19 | Uncertainty  Recovery | Not covered |  |  |  |
| *T03:"I simply write things down, like using notes in my phone or just like a notepad. So I can remind myself, but sometimes i forgot”* | TBI | Using compensatory strategies (notepad, phone) helpful for memory loss | Uncertainty Cognition  Symptom management | Not covered  Body Function | b1 Mental Functions | b144 Memory Functions |  |
| *T03:“I write things down. Basically I write everything down. If it's something I need to remember I write it down"* | TBI | Compensatory strategies like writing down info helps; memory deficits have an impact | Uncertainty Cognition  Symptom management | Not covered  Body Function | b1 Mental Functions | b144 Memory Functions |  |
| *T02:“I am watching my language so that my whole being part of myself self reflection has helped me easing myself talk. But i fail in doing that”* | TBI | Meta-cognitive strategies, and self-talk, monitoring thoughts and words helpful | Uncertainty Cognition  Symptom management | Not covered  Body Function | b1 Mental Functions | b144 Memory Functions |  |
| *T01:“I did just slow reintegration that was really helpful and I felt like I could involve myself,…I would do more that way that like I could remind myself that I still was able to do things, and I still remembered my protocols and I still know how to do my job"* | TBI | Slow, stepwise transition back to work was helpful | Uncertainty  Symptom management | Not covered |  |  |  |
| *T01: "when I felt good. I was obviously able to perform better, and like socialize more and do more of my daily activities"* | TBI | Uncertainty of impact of deficits; when doing well can participate, when doing poorly can participate less | Uncertainty  Recovery | Not covered |  |  |  |
| *T01: "But in general, my progress has been very slow and the main issue has been the slowness in getting back into my life"* | TBI | Speed of recovery and progress impacts mobility | Uncertainty  Recovery | Not covered |  |  |  |
| *T01: "I started going back in April, all the way, like I was progressive until November. It was a long journey back"* | TBI | Progress with recovery, access to rehab services helps with mobility | Uncertainty  Recovery | Not covered |  |  |  |
| *T05:“i ask myself if i would ever return normal and would my symptoms last for a life time. They recently told me to at xxx that my physiotherapy sessions have ended”* | TBI | Uncertainty of deficits and recovery limits mobility; lack of transition to community-based services | Uncertainty Symptoms Recovery | Not covered |  |  |  |
| *T03:“will I get relief from the symptoms? Given that they're being caused by two different things like perhaps the brain injury will get better. But, well, I still have the symptoms as a result of the eye damage”* | TBI | Uncertainty of deficits and recovery limits mobility | Uncertainty  Symptoms  Recovery | Not covered |  |  |  |
| *T03:"the symptoms are affecting me in a life changing way because I don't know how I'm going to do any kind of job Especially with The vertigo as well"* | TBI | Combination of deficits; uncertainty of progress and return to vocation | Uncertainty  Symptoms  Recovery | Not covered |  |  |  |
| *T01:I’m not sure if this is what you mean but I found myself asking all my therapists (e.g. physio) how long will these symptoms last ? no one could give a timeline”* | TBI | Lack of timeline for recovery difficult | Uncertainty  Recovery | Not covered |  |  |  |

^C: clinician; ICF: International Classification of Functioning, Disability and Health; S: stroke; T & TBI: traumatic brain injury^
