# Supplementary material for: Clinicians and individuals with acquired brain injury perspectives about factors that influence mobility: creating a core set of mobility domains among individuals with acquired brain injury
Source: Ann Med. 2021 Dec 13;53(1):2365–79. doi: 10.1080/07853890.2021.2015539 (PMC8676689; doi:10.1080/07853890.2021.2015539)
Supplement: Supplemental Material [file IANN_A_2015539_SM7437.zip › Supplemental files/Appendix 2.docx]

**Appendix 2. Inventory list of mobility measures**

| **Mobility measures** | **International Classification of Functioning, Disability and Health Component** |
| --- | --- |
| Two meter walking test | Activity and Participation |
| Five times sit to stand | Activity and Participation |
| Berg Balance Scale | Activity and Participation |
| Bilan articulaire + croix de Maigne | Activity and Participation |
| Community balance and mobility | Activity and Participation |
| Doigt-nez-, LEMOCOT, diadocokinésie | Body Function |
| Equitest-Motor Control Test | Activity and Participation |
| Flexibilité musculaire | Body Function |
| Fregly Ataxia Battery | Body Function |
| Manual muscle testing | Activity and Participation |
| Sensation : toucher léger/discrimination pique-touche, vibration, proprioception, température | Body Function |
| Tests de l’évaluation vestibulaire (inclus plusieurs tests) | Body Function |
| Timed- Stairs | Activity and Participation |
| Vitesses de marche (10-meter walking test) | Activity and Participation |
| Assis à debout 5X | Activity and Participation |
| Autonomie fonctionnelle - Chedoke-McMaster Sroke Assessment-Inventaire d’activités (CMSA-IA) | Activity and Participation |
| Coordination: locomotion (Lower Extremity Motor Coordination Test) | Body Function |
| Functional independence measure : Section locomotion | Activity and Participation |
| Force de préhension – dynamomètre manuel | Activity and Participation |
| Force musculaire – évaluation musculaire manuelle selon la méthode Daniels and Worthingham | Activity and Participation |
| Function in sitting test (FIST) | Activity and Participation |
| Grille d’évaluation des risques de chute en réadaptation, utilisée à l’interne, inspirée de la Morse Fall Scale. | Activity and Participation |
| Nottingham Sensory Assessment révisé (EmNSA) | Activity and Participation |
| Questionnaire sur la santé du patient (QSP-9) | Activity and Participation |
| Stade moteur du bras, de la main, de la jambe et du pied – Chedoke-McMaster Stroke Assessment-Inventaire des déficiences (CMSA-ID) | Activity and Participation |
| Step Test | Activity and Participation |
| Vitesse de marche : naturelle et rapide | Activity and Participation |
| BESTest (inclus le Mini-BESTest) | Activity and Participation |
| Capacité de marche fonctionnelle : Test de marche six minutes | Activity and Participation |
| Modified Ashworth Scale | Body Function |
| Rating of perceived exertion (Borg scale) | Body Function |
| Balance Master (Sensory Organization Test) | Body Function |
| Four Square Step Test | Activity and Participation |
| Stade de douleur à l’épaule hémiparétique – Chedoke-McMaster Stroke Assessment (CMSA) | Activity and Participation |
| Timed up and go test | Activity and Participation |
| Visual analogue scale-douleur | Body Function |
| Six minute walking test | Activity and Participation |
| Disabilities of the Arm, Shoulder and Hand questionnaire | Activity and Participation |
| Dyspnoea handicap inventory | Body Function |
| Minnesota manual dexterity test | Activity and Participation |
| Go outside and observation; real life, in authentic situation | Activity and Participation |
| Wheelchair skill assessment | Activity and Participation |
| Box and Block test | Activity and Participation |
| Functional independence measure | Activity and Participation |
| Capacity assessment | Activity and Participation |
| Energy expenditure | Activity and Participation |
| Useful Field of View (UFOV) | Body Function |
| Smith hand function evaluation | Activity and Participation |
| Presence of pain; Pain intensity; Stability of the person’s medical condition; Type of pain and Practice setting (PPSTP) | Body Function |
