# Supplementary material for: Clinicians and individuals with acquired brain injury perspectives about factors that influence mobility: creating a core set of mobility domains among individuals with acquired brain injury
Source: Ann Med. 2021 Dec 13;53(1):2365–79. doi: 10.1080/07853890.2021.2015539 (PMC8676689; doi:10.1080/07853890.2021.2015539)
Supplement: Supplemental Material [file IANN_A_2015539_SM7437.zip › Supplemental files/Appendix 3.docx]

**Appendix 3. Thematic content analysis based on the coding rating (the frequency of each code within each theme among all participants)**

| **Codes** | **All participants (n=27) n (%)** | **Clinicians (n=17) n (%)** | **Patients (n=10) n (%)** |
| --- | --- | --- | --- |
| **Theme 1: Considering mobility holistically and individuals needs, preferences, and unique experiences** | | | |
| **1.1. A comprehensive definition of Mobility** | | | |
| - Cognition | 4 (14%) | 2 (12%) | 2 (20%) |
| - Walking | 4 (14%) |  | 4 (40%) |
| - Anxiety | 3 (11%) | 2 (12%) | 1 (10%) |
| - Safety | 3 (11%) | 3 (17%) |  |
| - Work | 2 (7%) | 1 (6%) | 1 (10%) |
| - Fear | 2 (7%) | 2 (12%) |  |
| - Leisure activities | 2 (7%) | 2 (12%) |  |
| - Finances | 1 (3%) |  | 1 (10%) |
| - Behaviour | 1 (3%) | 1 (6%) |  |
| - Biking | 1 (3%) | 1 (6%) |  |
| - Community re-integration | 1 (3%) | 1 (6%) |  |
| - Driving | 1 (3%) |  | 1 (10%) |
| - Motivation | 1 (3%) | 1 (6%) |  |
| - Physical capacity | 1 (3%) | 1 (6%) |  |
| - Psychological | 1 (3%) |  | 1 (10%) |
| - Relationship with family | 1 (3%) |  | 1 (10%) |
| - Self-confidence | 1 (3%) | 1 (6%) |  |
| - Self-efficacy | 1 (3%) | 1 (6%) |  |
| - Self-identity | 1 (3%) |  | 1 (10%) |
| - Self-isolation | 1 (3%) |  | 1 (10%) |
| - Social life | 1 (3%) |  | 1 (10%) |
| - Travel | 1 (3%) |  | 1 (10%) |
| **1.2. Factors hindering mobility, participation and reintegration into the community** | | | |
| - Cognition | 5 (18%) | 1 (6%) | 4 (40%) |
| - Fatigue | 4 (14%) |  | 4 (40%) |
| - Headache | 4 (14%) |  | 4 (40%) |
| - Self-identity | 3 (11%) |  | 3 (30%) |
| - Dizziness | 3 (11%) | 1 (6%) | 2 (20%) |
| - Fear | 3 (11%) | 1 (6%) | 2 (20%) |
| - Emotions | 2 (7%) |  | 2 (20%) |
| - Nausea | 2 (7%) |  | 2 (20%) |
| - Balance | 1 (3%) | 1 (6%) |  |
| - Confidence | 1 (3%) | 1 (6%) |  |
| - Confusion | 1 (3%) | 1 (6%) |  |
| - Depression | 1 (3%) |  | 1 (10%) |
| - Double vision | 1 (3%) |  | 1 (10%) |
| - Fall | 1 (3%) | 1 (6%) |  |
| - Finding words/Speech | 1 (3%) |  | 1 (10%) |
| - Hallucination | 1 (3%) |  | 1 (10%) |
| - Independence | 1 (3%) |  | 1 (10%) |
| - Physical | 1 (3%) |  | 1 (10%) |
| - Psychological | 1 (3%) |  | 1 (10%) |
| - Relationship with family | 1 (3%) |  | 1 (10%) |
| - Safety | 1 (3%) | 1 (6%) |  |
| - Sleep disturbance | 1 (3%) |  | 1 (10%) |
| - Loss of driving license | 1 (3%) |  | 1 (10%) |
| **1.3. Impacts of bio-psychosocial factors on everyday life and mobility** | | | |
| - Cognitive | 8 (29%) |  | 8 (80%) |
| - Work | 6 (22%) |  | 6 (60%) |
| - Social life | 5 (18%) |  | 5 (50%) |
| - Reading/comprehension | 4 (14%) |  | 4 (40%) |
| - Sensitivity to stimulation | 4 (14%) |  | 4 (40%) |
| - Leisure activities | 3 (11%) |  | 3 (30%) |
| - Auditory | 2 (7%) |  | 2 (20%) |
| - Family relationship | 2 (7%) |  | 2 (20%) |
| - Psychological | 2 (7%) |  | 2 (20%) |
| - Self-isolation | 2 (7%) |  | 2 (20%) |
| - Visual | 2 (7%) |  | 2 (20%) |
| - Driving | 2 (7%) |  | 2 (20%) |
| - Fear | 1 (3%) |  | 1 (10%) |
| - Anxiety | 1 (3%) |  | 1 (10%) |
| - Depression | 1 (3%) |  | 1 (10%) |
| - Fatigue | 1 (3%) |  | 1 (10%) |
| - Financial | 1 (3%) |  | 1 (10%) |
| - Self-identity | 1 (3%) |  | 1 (10%) |
| - Sport | 1 (3%) |  | 1 (10%) |
| - Weather | 1 (3%) |  | 1 (10%) |
| **Theme 2: Assessment and intervention guidelines** | | | |
| **2.1 Finding common goals with patients** | | | |
| - Clinical judgment Standardized measures | 11 (40%) | 11 (65%) |  |
| - Interdisciplinary shared decision making | 10 (37%) | 10 (59%) |  |
| - Self-reported Screening | 10 (37%) | 10 (59%) |  |
| - Situational/Observation | 9 (33%) | 9 (53%) |  |
| - Clinical judgement Expertise | 8 (29%) | 8 (47%) |  |
| - Patient objective Clinical judgment | 8 (29%) | 8 (47%) |  |
| - Cognition/Aphasia | 6 (22%) | 6 (35%) |  |
| - Self-reported/limited | 4 (14%) | 4 (23%) |  |
| - Screening Assessment | 4 (14%) | 4 (23%) |  |
| - Standardized measures /responsiveness | 2 (7%) | 2 (12%) |  |
| - Red flag indicators | 2 (7%) | 2 (12%) |  |
| - Functional capacity | 1 (3%) | 1 (6%) |  |
| - Standardized measures /consistency | 1 (3%) | 1 (6%) |  |
| - Standardized measures /global and recommendation | 1 (3%) | 1 (6%) |  |
| - Proxy assessment | 1 (3%) | 1 (6%) |  |
| - Balance | 1 (3%) | 1 (6%) |  |
| - Driving | 1 (3%) | 1 (6%) |  |
| - Coordination | 1 (3%) | 1 (6%) |  |
| - Pain | 1 (3%) | 1 (6%) |  |
| - Endurance | 1 (3%) | 1 (6%) |  |
| - Safety | 1 (3%) | 1 (6%) |  |
| - Spasticity | 1 (3%) | 1 (6%) |  |
| - Strength | 1 (3%) | 1 (6%) |  |
| - Vision | 1 (3%) | 1 (6%) |  |
| **2.2. Challenges clinicians faced when they evaluate mobility** | | | |
| - Standardized measures/ limited | 9 (33%) | 9 (53%) |  |
| - Safety | 8 (29%) | 8 (47%) |  |
| - Cognitive | 7 (26%) | 7 (41%) |  |
| - Confidence | 3 (11%) | 3 (17%) |  |
| - Guidelines | 2 (7%) | 2 (12%) |  |
| - Alcohol/Drug | 2 (7%) | 2 (12%) |  |
| - Weather | 2 (7%) | 2 (12%) |  |
| - Anxiety | 1 (3%) | 1 (6%) |  |
| - Balance | 1 (3%) | 1 (6%) |  |
| - Fatigue | 1 (3%) | 1 (6%) |  |
| - Fear | 1 (3%) | 1 (6%) |  |
| - Trust | 1 (3%) | 1 (6%) |  |
| - Vision | 1 (3%) | 1 (6%) |  |
| **2.3. Engaging the patient and considering their perspectives in their care** | | | |
| - Patient engagement | 7 (26%) |  | 7 (70%) |
| **Theme 3: Support network** | | | |
| **3.1. Caregiver support** | | | |
| - Caregiver | 13 (48%) | 5 (29%) | 8 (80%) |
| - Cognition | 4 (14%) | 3 (17%) | 1 (10%) |
| - Aphasia | 1 (3%) | 1 (6%) |  |
| - Mental health | 1 (3%) |  |  |
| **3.2. Provider support** | | | |
| - Provider | 3 (11%) |  | 3 (30%) |
| - Family support | 1 (3%) | 1 (6%) |  |
| - Coping style | 1 (3%) | 1 (6%) |  |
| **3.3. Community support** | | | |
| - Community | 8 (29%) | 4 (23%) | 4 (40%) |
| - Stigma | 2 (7%) |  | 2 (20%) |
| **Theme 4: Uncertainty about symptoms and recovery** | | | |
| - Recovery | 4 (14%) |  | 4 (40%) |
| - Symptom management strategies | 4 (14%) |  | 4 (40%) |
| - Symptoms | 3 (11%) |  | 3 (30%) |
| - Cognition | 3 (11%) |  | 3 (30%) |
| - Psychological | 1 (3%) |  | 1 (10%) |
